# *Synechocystis* KaiC3 Displays Temperature- and KaiB-Dependent ATPase Activity and Is Important for Growth in Darkness

**DOI:** 10.1128/JB.00478-19

**Published:** 2020-01-29

**Authors:** Anika Wiegard, Christin Köbler, Katsuaki Oyama, Anja K. Dörrich, Chihiro Azai, Kazuki Terauchi, Annegret Wilde, Ilka M. Axmann

**Affiliations:** a Institute for Synthetic Microbiology, Cluster of Excellence on Plant Sciences (CEPLAS), Heinrich Heine University Duesseldorf, Duesseldorf, Germany; b Institute of Biology III, Faculty of Biology, University of Freiburg, Freiburg, Germany; c Graduate School of Life Sciences, Ritsumeikan University, Kusatsu, Shiga, Japan; d Institute for Microbiology and Molecular Biology, Justus Liebig University, Giessen, Germany; e College of Life Sciences, Ritsumeikan University, Kusatsu, Shiga, Japan; Université de Montréal

**Keywords:** ATPase, KaiC, *Synechocystis*, circadian clock, cyanobacteria, photosynthetic bacteria

## Abstract

Cyanobacteria form a heterogeneous bacterial group with diverse lifestyles, acclimation strategies, and differences in the presence of circadian clock proteins. In Synechococcus elongatus PCC 7942, a unique posttranslational KaiABC oscillator drives circadian rhythms. ATPase activity of KaiC correlates with the period of the clock and mediates temperature compensation. *Synechocystis* sp. strain PCC 6803 expresses additional Kai proteins, of which KaiB3 and KaiC3 proteins were suggested to fine-tune the standard KaiAB1C1 oscillator. In the present study, we therefore characterized the enzymatic activity of KaiC3 as a representative of nonstandard KaiC homologs *in vitro*. KaiC3 displayed ATPase activity lower than that of the Synechococcus elongatus PCC 7942 KaiC protein. ATP hydrolysis was temperature dependent. Hence, KaiC3 is missing a defining feature of the model cyanobacterial circadian oscillator. Yeast two-hybrid analysis showed that KaiC3 interacts with KaiB3, KaiC1, and KaiB1. Further, KaiB3 and KaiB1 reduced *in vitro* ATP hydrolysis by KaiC3. Spot assays showed that chemoheterotrophic growth in constant darkness is completely abolished after deletion of Δ*kaiAB1C1* and reduced in the absence of *kaiC3*. We therefore suggest a role for adaptation to darkness for KaiC3 as well as a cross talk between the KaiC1- and KaiC3-based systems.

**IMPORTANCE** The circadian clock influences the cyanobacterial metabolism, and deeper understanding of its regulation will be important for metabolic optimizations in the context of industrial applications. Due to the heterogeneity of cyanobacteria, characterization of clock systems in organisms apart from the circadian model Synechococcus elongatus PCC 7942 is required. *Synechocystis* sp. strain PCC 6803 represents a major cyanobacterial model organism and harbors phylogenetically diverged homologs of the clock proteins, which are present in various other noncyanobacterial prokaryotes. By our *in vitro* studies we unravel the interplay of the multiple *Synechocystis* Kai proteins and characterize enzymatic activities of the nonstandard clock homolog KaiC3. We show that the deletion of *kaiC3* affects growth in constant darkness, suggesting its involvement in the regulation of nonphotosynthetic metabolic pathways.

## Abstract (precis)

The circadian clock influences the cyanobacterial metabolism, and deeper understanding of its regulation will be important for metabolic optimizations in the context of industrial applications. Due to the heterogeneity of cyanobacteria, characterization of clock systems in organisms apart from the circadian model Synechococcus elongatus PCC 7942 is required. *Synechocystis* sp. strain PCC 6803 represents a major cyanobacterial model organism and harbors phylogenetically diverged homologs of the clock proteins, which are present in various other noncyanobacterial prokaryotes. By our *in vitro* studies we unravel the interplay of the multiple *Synechocystis* Kai proteins and characterize enzymatic activities of the nonstandard clock homolog KaiC3. We show that the deletion of *kaiC3* affects growth in constant darkness, suggesting its involvement in the regulation of nonphotosynthetic metabolic pathways.

## INTRODUCTION

Cyanobacteria have evolved the circadian clock system to sense, anticipate, and respond to predictable environmental changes based on the rotation of the Earth and the resulting day-night cycle. Circadian rhythms are defined by three criteria: (i) oscillations with a period of about 24 h without external stimuli, (ii) synchronization of the oscillator with the environment, and (iii) compensation for the usual temperature dependence of biochemical reactions, so that the period of endogenous oscillation does not depend on temperature in a physiological range (1). The cyanobacterial circadian clock system has been studied in much detail in the unicellular model cyanobacterium Synechococcus elongatus PCC 7942 (here *S. elongatus*). Its core oscillator is composed of three proteins, which are unique to prokaryotes: KaiA, KaiB, and KaiC (note that some of the information cited here was gained by studying proteins from Thermosynechococcus elongatus BP-1) (2). The level of KaiC phosphorylation and KaiC’s ATPase activity represent the key features of the biochemical oscillator. KaiA stimulates autophosphorylation and ATPase activity of KaiC, whereas KaiB binding to the complex inhibits KaiA action, stimulates autodephosphorylation activity, and reduces ATPase activity of KaiC (3–5). As a consequence of dynamic interactions with KaiA and KaiB, KaiC rhythmically phosphorylates and dephosphorylates with a 24-h period (2).

KaiC consists of two replicated domains (CI and CII) that assemble into a hexamer, forming an N-terminal CI ring and a C-terminal CII ring (6–8). Phosphorylation takes place in the CII ring (9), whereas ATP hydrolysis occurs in both rings (10). In the CII ring, ATP hydrolysis is part of the dephosphorylation mechanism (11, 12). ATP hydrolysis in the CI ring correlates with the period of the clock and temperature compensation and is further required for a conformational change of KaiC, which allows binding of KaiB (4, 13, 14). The levels of phosphorylation and ATP hydrolysis of KaiC serve as the readout for regulatory proteins, which orchestrate the circadian output (15, 16). For a recent review on the functioning of the KaiABC system, see Swan et al. (17).

In a natural day and night cycle, *S. elongatus* orchestrates its metabolism on a precise temporal schedule, with the metabolism being adjusted by the clock but also feeding input information to the clock (18–20). Knockout of the *S. elongatus kai* genes leads to a growth disadvantage. When grown in competition with the wild type under light-dark conditions, the clock-deficient mutant cells are eliminated from the culture (21). Growth in light-dark cycles is also impaired by deletion of the regulator of phycobilisome association A (RpaA) (18), the master transcription factor of circadian gene expression. In addition, studies on single-nucleotide polymorphisms identified the *rpaA* gene as one of three genes responsible for the faster growth of Synechococcus elongatus UTEX 2973 than *S. elongatus* (23). Using a transposon library, Welkie et al. (24) showed that KaiA, despite being nonessential for growth in light-dark cycles, strongly contributes to the fitness of *S. elongatus* under these conditions. Decreased fitness most likely occurs due to reduced phosphorylation of RpaA in the *kaiA* knockout strain (24). Overall, these data demonstrate the value of the cyanobacterial clock system for metabolic orchestration under natural conditions, with the clock system considered especially important for the transition from light to darkness (25).

Cyanobacteria represent one of the most diverse prokaryotic phyla (26), and little is known about timekeeping mechanisms in cyanobacteria other than *S. elongatus*. A core diurnal genome has been described (27), but temporal coordination varies, and based on genomic analyses, large variations in the cyanobacterial clock systems can be expected (28–33).

The cyanobacterial model strain *Synechocystis* sp. strain PCC 6803 (here called *Synechocystis*) contains a standard *kai* operon, encoding homologs of the *kaiA*, *kaiB*, and *kaiC* genes (here designated *kaiA_6803_*, *kaiB1*, and *kaiC1*) as well as two additional copies each of *kaiB* and *kaiC* (designated *kaiB2* and *kaiB3* and *kaiC2* and *kaiC3*) (34). Note that naming of KaiC3/KaiC2 and KaiB3/KaiB2 is not consistent in the literature. In this paper, we name the *Synechocystis kai* genes according to Aoki and Onai, Wiegard et al., and Schmelling et al. (27, 29, 31). The *kaiC2* and *kaiB2* genes form an operon, whereas *kaiC3* and *kaiB3* are orphan genes. Based on phylogenetic reconstruction analysis, the KaiB and KaiC proteins of *Synechocystis* were allocated in three phylogenetically different subclasses (31, 35). KaiC1 and KaiB1 display 82% and 88% amino acid identity to *S. elongatus* KaiC and KaiB, respectively. Amino acid identity to *S. elongatus* proteins is lower for the other *Synechocystis* Kai homologs, namely, 37% for KaiC2, 52% for KaiB2, 55% for KaiC3, and 48% for KaiB3. The KaiC homologs show differences in their C termini: both KaiC2 and KaiC3 show low conservation of the A-loop. KaiC2 further differs from KaiC3 by displaying modified phosphorylation sites (two serine residues instead of serine and threonine in KaiC1 and KaiC3) (31).

Nevertheless, all three KaiC proteins from *Synechocystis* show high conservation of the kinase motif in the CII domain, and all three proteins were shown to exhibit autophosphorylation activity (27, 31). KaiA_6803_ stimulates the autophosphorylation of KaiC1 but does not affect the phosphorylation of the two other KaiC homologs. Based on sequence analysis and experimental validation of kinase activity, Schmelling et al. (27) concluded that autokinase and autophosphatase activities are highly conserved features of all cyanobacterial KaiC homologs. Likewise, ATPase motifs in the N-terminal CI domain of KaiC are highly conserved in all cyanobacterial KaiC homologs (27). However, ATP hydrolysis was only characterized for several KaiC1 homologs and a KaiC2 homolog from Rhodopseudomonas palustris (10, 36–38).

In *Synechocystis*, deletion of the *kaiAB1C1* gene cluster causes reduced growth in light-dark rhythms, even when grown as a single culture, demonstrating a more pronounced phenotype than *S. elongatus*, where only a competitive growth disadvantage was observed (39). Deletion of *rpaA* also reduced growth in light-dark cycles, which further implies metabolic orchestration by the *Synechocystis* timer (22). The reported number of oscillating genes in *Synechocystis* varies largely among different studies, which is likely due to differences in growth conditions and strain variations (40–43). Aoki and Onai (29) suggested that KaiC3 and KaiB3 modulate the amplitude and period of the KaiAB1C1 oscillator, whereas disruption of *kaiC2B2* implied a non-clock-related function for KaiC2 and KaiB2. There are different *Synechocystis* laboratory strain variants in use, which show phenotypic variation, for example, in their glucose tolerance, motility, and tolerance to abiotic stress (44). In the *Synechocystis* strain used in this study (PCC-M, resequenced [45]), the *kaiC2B2* cluster cannot be deleted (39), which further implies a non-clock-related essential function. The function of KaiC3 has not been addressed so far. BLAST analysis identified KaiC3 homologs to be present in addition to KaiC1 in about one-third of cyanobacterial genera. KaiC3 homologs were also found in noncyanobacterial eubacteria and archaea, where they show a higher distribution than the phylogenetically different KaiC1 and KaiC2 homologs (27).

In this study, we therefore aim at a detailed biochemical characterization of the putative clock component KaiC3 and the role of *Synechocystis* KaiB homologs in modulation of KaiC3 function. Characterization of the ATPase activity of KaiC3 was of special interest, because ATP hydrolysis defines the period length and temperature compensation of the Kai oscillator (4). We further demonstrate that the Δ*kaiC3* mutant strain has a growth defect under chemoheterotrophic growth conditions, which is similar to but less pronounced than those of Δ*kaiAB1C1* and Δ*rpaA* strains (22, 39). Our data support the idea of a function of KaiC3 and KaiB3 in fine-tuning the central oscillator composed of KaiA_6803_, KaiB1, and KaiC1 in *Synechocystis*.

(This article was submitted to an online preprint archive [46].)

## RESULTS

### Recombinant KaiC3 displays only low ATP synthase activity.

Previous bioinformatic analysis predicted that kinase, dephosphorylation, and ATPase activities are conserved in KaiC3 (27). So far, only the kinase activity of KaiC3 was experimentally confirmed, and it differed from the activity of *S. elongatus* KaiC by lacking stimulation by KaiA and KaiA_6803_ (31). Therefore, we aimed to characterize further predicted activities of KaiC3.

**FIG 1:**
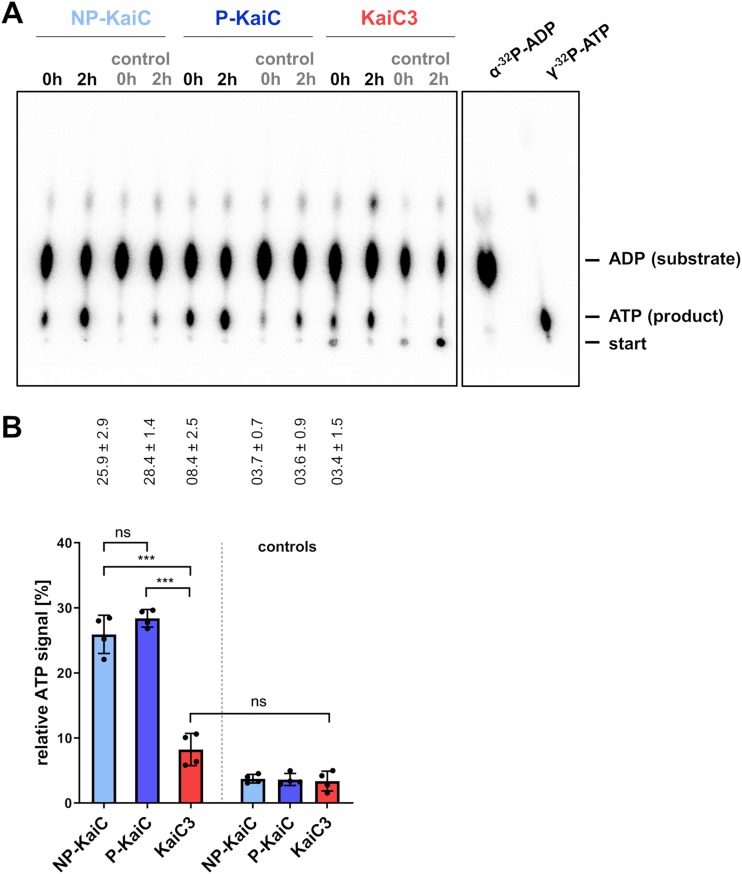
KaiC3 showed no or highly decreased ATP synthase activity compared to that of KaiC. Prior to the experiment, KaiC was incubated for 2 weeks at 4°C or overnight at 30°C to generate fully phosphorylated (P-KaiC) and dephosphorylated (NP-KaiC) protein (Fig. S1). (A) Representative autoradiograph of separation of [α-^32^P]ATP (product) and [α-^32^P]ADP (substrate) via thin-layer chromatography after incubation with indicated KaiC proteins for 2 h. Controls show the ATP signal in the presence of an excess of cold ADP. For size control, [γ-^32^P]ATP and [α-^32^P]ADP were separated on the same cellulose F plate. (B) Relative ATP signals after 2 h of incubation, displayed as a percentage of all radioactive signals in the corresponding lane (means with standard deviations from two experiments, each analyzed in duplicates). All values are normalized to the relative ATP signal at 0 h of incubation time in the presence of an excess of cold ADP. Statistical significance was calculated by Brown-Forsythe and Welch analysis of variance (ANOVA) tests, followed by Dunnett’s T3 multiple-comparison test using GraphPad Prism 8. Significance of the mean difference is indicated as nonsignificant (*P* ≥ 0.05, ns), significant with *P* = 0.01 to 0.05 (*), very significant with *P* = 0.001 to 0.01 (**), extremely significant with *P* = 0.0001 to 0.001 (***), and extremely significant with *P* < 0.0001 (****).

The first step in KaiC dephosphorylation is regarded as a reversal of phosphorylation: KaiC transfers its bound phosphoryl group to ADP and thereby synthesizes ATP (11, 12). To investigate reversibility of intrinsic phosphorylation, we tested whether KaiC3 can synthesize [α-^32^P]ATP from [α-^32^P]ADP (Fig. 1). As a control, we used phosphorylated and dephosphorylated KaiC (see Fig. S1 in the supplemental material). Immediately after adding [α-^32^P]ADP, all KaiC proteins started to synthesize [α-^32^P]ATP. Phosphorylated as well as nonphosphorylated KaiC showed higher initial ATP synthesis than KaiC3 (Fig. 1). After incubating phosphorylated and dephosphorylated KaiC for 2 h at 30°C with [α-^32^P]ADP, a relative [α-^32^P]ATP level of 26 to 28% was detected. KaiC3 showed low intrinsic ATP synthesis, but formation of relative [α-^32^P]ATP was not significantly higher than that in the control with cold ATP. This can be explained by two hypotheses: (i) compared to *S. elongatus* KaiC, KaiC3 showed lower phosphotransferase activity or (ii) higher consumption of the produced ATP. The latter would require that KaiC3 exhibits higher ATPase activity than KaiC. Therefore, we next examined ATP hydrolysis activity of KaiC3.

### KaiC3 displays ATPase activity.

**FIG 2:**
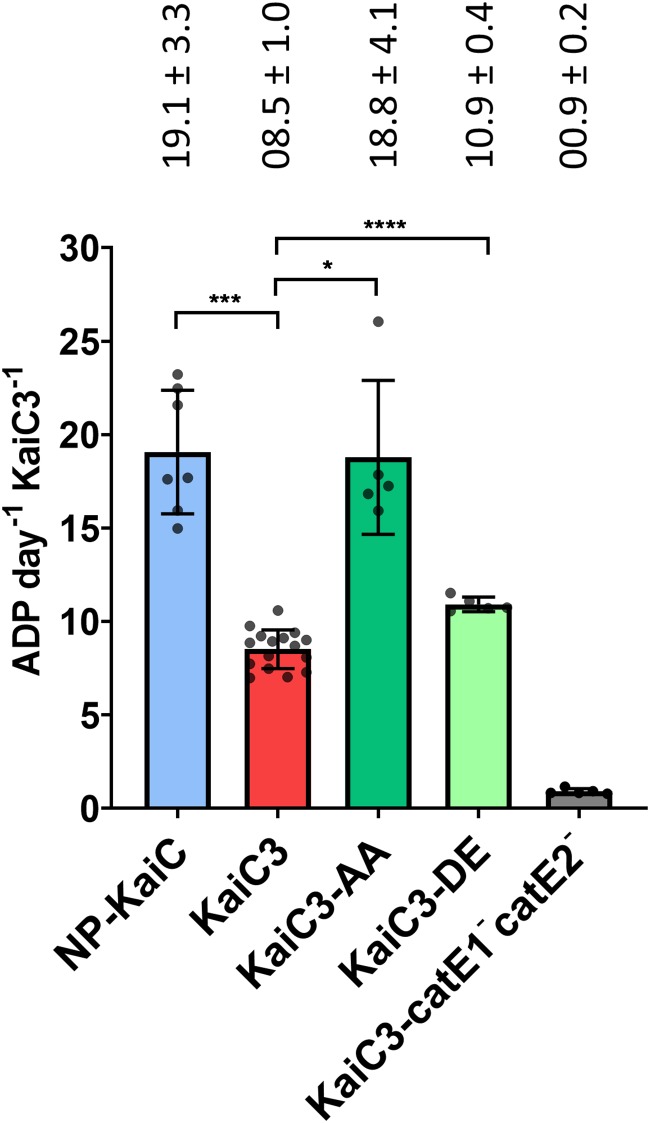
ATPase activity of Strep-KaiC3 and variants Strep-KaiC3-DE and Strep-KaiC3-AA. Strep-KaiC3, Strep-KaiC3-DE, and Strep-KaiC3-AA were incubated for 24 h at 30°C, and ADP production per day and per Strep-KaiC3 monomer was calculated. As a control we used Strep-KaiC3-catE1-catE2-, which lacks both ATPase motifs due to replacement of catalytic glutamates 67, 68, 310, and 311 with glutamine residues. For comparison, ADP production by KaiC was monitored for 24 or 48 h. Strep-KaiC was dephosphorylated prior to the experiment by incubation at 30°C overnight. Shown are mean values with standard deviations from at least five replicates. Statistical significance was calculated by Brown-Forsythe and Welch ANOVA tests, followed by Dunnett’s T3 multiple-comparison test using GraphPad Prism 8. Significance of the mean difference is indicated as significant with *P* = 0.01 to 0.05 (*), extremely significant with *P* = 0.0001 to 0.001 (***), and extremely significant with *P* < 0.0001 (****). Note that we averaged all measurements for Strep-KaiC3 WT at 30°C and, therefore, display them again in Fig. 4A and B.

ATP hydrolysis can be measured by quantifying ADP production by KaiC over time (4). Conservation of WalkerA and WalkerB motifs in the CI domain of KaiC3 proteins suggested their ability to hydrolyze ATP (27). We purified recombinant hexameric KaiC3 wild-type protein fused to an N-terminal Strep-tag (Strep-KaiC3) (Fig. S4) and confirmed the predicted ATPase activity *in vitro*. Based on the measured bulk ATPase activity, we calculated that 8.5 ± 1.0 ADP molecules per monomer and day (means ± standard deviations [SD]) (Fig. 2) were produced by Strep-KaiC3. We were not able to purify stable recombinant KaiC1 with sufficient purity and therefore used the highly similar KaiC ortholog from *S. elongatus* for comparison. The ATPase activity of Strep-KaiC3 was about 45% of the value for KaiC from *S. elongatus* (19.1 ± 3.3 ADP molecules per day). Hence, the above-observed lower level of net ATP production by KaiC3 was not based on a higher ATP consumption but due to lower dephosphorylation activity *per se*. In KaiC, the ATPase activity changes after substitution of the phosphorylation sites (4). We therefore generated variants of Strep-KaiC3 in which residues S423 and T424 are either replaced with aspartate and glutamate (Strep-KaiC3-DE) or replaced with two alanine residues (Strep-KaiC3-AA). Strep-KaiC3-AA showed more than 2-fold increased ATPase activity, whereas ATP hydrolysis by Strep-KaiC3-DE was only slightly different from that of the wild-type protein (Fig. 2) and not reduced as reported for *S. elongatus* KaiC-DE (4).

### KaiC3 interacts with KaiB3 and components of the standard KaiAB1C1 oscillator.

**FIG 3:**
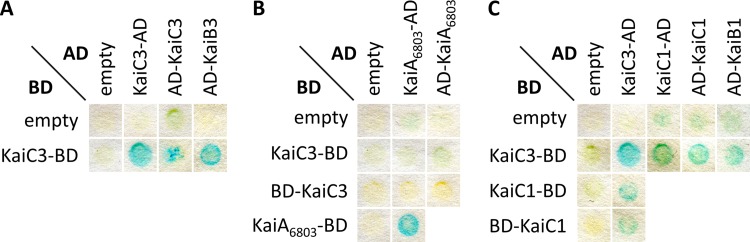
KaiC3 interacts with KaiB3 and the proteins of the main oscillator KaiC1, KaiB1. Yeast two-hybrid reporter strains carrying bait and prey plasmids were selected by plating on complete supplement medium (CSM) lacking leucine and tryptophan (−Leu −Trp). Physical interaction between bait and prey fusion proteins is indicated by a color change in the assays using 5-bromo-4-chloro-3-indoxyl-β-d-galactopyranoside. AD, GAL4 activation domain; BD, GAL4 DNA-binding domain. Shown are representative results from two replicates. For clear presentation, spots were assembled from several assays (original scans are shown in Fig. S2).

*In vitro* and *in silico* studies suggested that KaiA_6803_, KaiB1, and KaiC1 form the standard clock system of *Synechocystis* (29, 31). Since Aoki and Onai (29) suggested that KaiC3 modulates the main oscillator function, we performed protein-protein interaction studies in order to reveal a possible cross talk between the multiple Kai proteins. First, interaction was determined by yeast two-hybrid analysis using KaiC1, KaiC3, KaiB1, and KaiB3 fused to AD and BD domains, respectively. The color change of the colonies indicates β-galactosidase activity and is an estimate for the interaction of the two proteins. As expected, these experiments showed self-interaction of KaiC3 (Fig. 3A), since KaiC homologs are known to form hexamers (6, 8). In addition, an interaction of KaiC3 with KaiB3 was detected (Fig. 3A), which is in line with bioinformatic analysis showing frequent cooccurrence of *kaiC3* and *kaiB3* genes in genomes (27). We could not detect an interaction between KaiC3 and KaiA_6803_ by yeast two-hybrid analysis (Fig. 3B) but detected a heteromeric interaction between KaiC1 and KaiC3 using different protein fusion variants (Fig. 3C). These results confirm previously published data, which showed (i) copurification of KaiA with KaiC1 but not with KaiC3 and (ii) a weak interaction between the two KaiC homologs KaiC1 and KaiC3 in *ex vivo* pulldown analysis followed by Western blot analysis (31). Besides KaiC1, the cognate KaiB protein KaiB1 also showed an interaction with KaiC3 in our analysis (Fig. 3C), corroborating the hypothesis that there is a cross talk between the putative KaiC3-B3 system and the core oscillator KaiAB1C1. Such a cross talk via the KaiB proteins was further supported by our *in vitro* pulldown assays, in which KaiC3 interacted with KaiB3 and further with KaiB1 (Fig. S3B and D). In addition, KaiC1 interacted with both KaiB1 and KaiB3 homologs (Fig. S3A and C). One must take into account that based on the *in vitro* pulldown assays using *Synechocystis* whole-cell extracts, we cannot exclude indirect interactions. As we have shown that KaiC1 and KaiC3 interact with each other, both proteins could bind as a heterohexamer to the glutathione *S*-transferase (GST)-tagged KaiB proteins and therefore coelute from the affinity matrix.

### ATPase activity of KaiC3 is reduced in the presence of KaiB1 and KaiB3.

**FIG 4:**
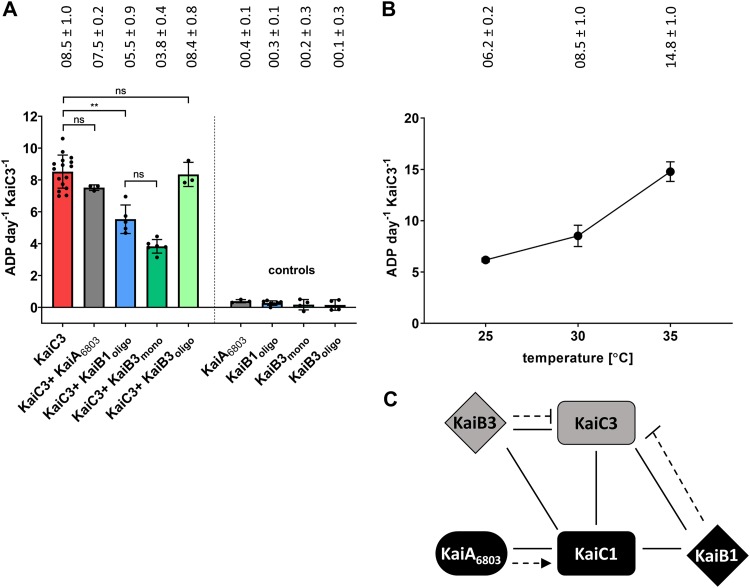
ATPase activity of Strep-KaiC3 is regulated by KaiB proteins and is temperature dependent. (A) ATPase activity of Strep-KaiC3 was decreased in the presence of KaiB1 and monomeric KaiB3 but was not influenced by oligomeric KaiB3 and KaiA_6803_. Shown are mean values with standard deviations from at least three replicates. Controls show that KaiA_6803_ and KaiB proteins alone did not display ATPase activity (referring to Strep-KaiC3 monomer activity). Statistical significance was calculated by Brown-Forsythe and Welch ANOVA tests, followed by Dunnett’s T3 multiple-comparison test using GraphPad Prism 8. Significance of the mean difference is indicated as nonsignificant (*P* ≥ 0.05; ns) or very significant with *P* = 0.001 to 0.01 (**). (B) ATPase activity of Strep-KaiC3 is temperature dependent. Strep-KaiC3 was incubated for 24 h at the indicated temperatures, and ADP production per day and per monomer Strep-KaiC3 was calculated. Shown are mean values with standard deviations from at least three replicates. A temperature-compensated activity would result in a flat horizontal line. Note that we averaged all measurements for Strep-KaiC3 WT at 30°C and, therefore, also display them in Fig. 2. (C) Summary of the cross talk between the KaiAB1C1 standard oscillator and KaiC3 in *Synechocystis*. We hypothesize that KaiC1 forms a standard oscillator together with KaiA_6803_ and KaiB1. KaiC3 might form an additional regulatory mechanism together with KaiB3. The two systems intertwine by interactions of the KaiC proteins with each other and the noncorresponding KaiB homologs. Solid lines indicate experimentally verified physical interactions (this paper and Wiegard et al. [31]), the dotted arrow indicates stimulation of phosphorylationdotted arrow indicates stimulation of phosphorylation (31), and dotted lines with bars show inhibition of ATPase activity.

The interaction of KaiC3 with KaiB1 and KaiB3 (Fig. 3 and Fig. S3) suggested a regulation of KaiC3 activity by these two KaiB proteins. We therefore measured ATP hydrolysis by Strep-KaiC3 in the presence of 0.04 mg ml^−1^ KaiB1 and KaiB3 proteins, respectively (Fig. 4A). After size exclusion chromatography, KaiB1 was only eluted as a tetramer (44 and 72 kDa, respectively, depending on the column), whereas KaiB3 was eluted as a monomer (13/23 kDa) and tetramer (41/70 kDa) (Fig. S4). Therefore, the monomeric and tetrameric KaiB3 fractions were tested separately. In all measurements, the ATPase activity was linear and showed no oscillations. ATP hydrolysis was reduced by 55% after the addition of the KaiB3 monomer but was not affected by the KaiB3 tetramer. The KaiB1 tetramer also inhibited the ATPase activity of Strep-KaiC3 (35% reduction compared to Strep-KaiC3 alone) (Fig. 4). These data imply a trend in which KaiB1 has less effect on KaiC3’s ATPase activity than the KaiB3 monomer, but the differences were not statistically significant (Fig. 4). Because ATPase activity of KaiC is influenced by KaiA (4), we also investigated the effect of KaiA_6803_ on KaiC3, but in line with the lack of interaction in our yeast two-hybrid analysis data (Fig. 3B), ATPase activity of Strep-KaiC3 was not significantly affected by KaiA_6803_ (Fig. 4A). Figure 4C summarizes the Kai protein interactions and regulation of KaiC3’s ATPase activity by KaiB proteins.

### ATPase activity of KaiC3 is temperature dependent.

True circadian clocks are characterized by temperature-compensated oscillations, which ensure robust time measurements under temperature fluctuations. In the *S. elongatus* KaiABC clock, overall temperature compensation is derived from KaiC’s ATPase activity, which is stable between 25 and 35°C (*Q*_10_ = 1.2 [4]). We therefore asked whether N-Strep-KaiC3, as a representative of nonstandard KaiC homologs, shows temperature compensation as well. Measurements at 25, 30, and 35°C, however, revealed a temperature-dependent ADP production by KaiC3 (*Q*_10_ = 2.4 [Fig. 4B]; activation energy, 66.5 kJ mol^−1^ [Fig. S5A]). Hence, KaiC3 is lacking a characteristic feature of circadian oscillators. In accordance, dephosphorylation of Strep-KaiC3 was higher at 25°C than at 30 and 35°C (Fig. S5B).

### The nonstandard KaiC3 protein supports growth of *Synechocystis* cells in the dark.

**FIG 5:**
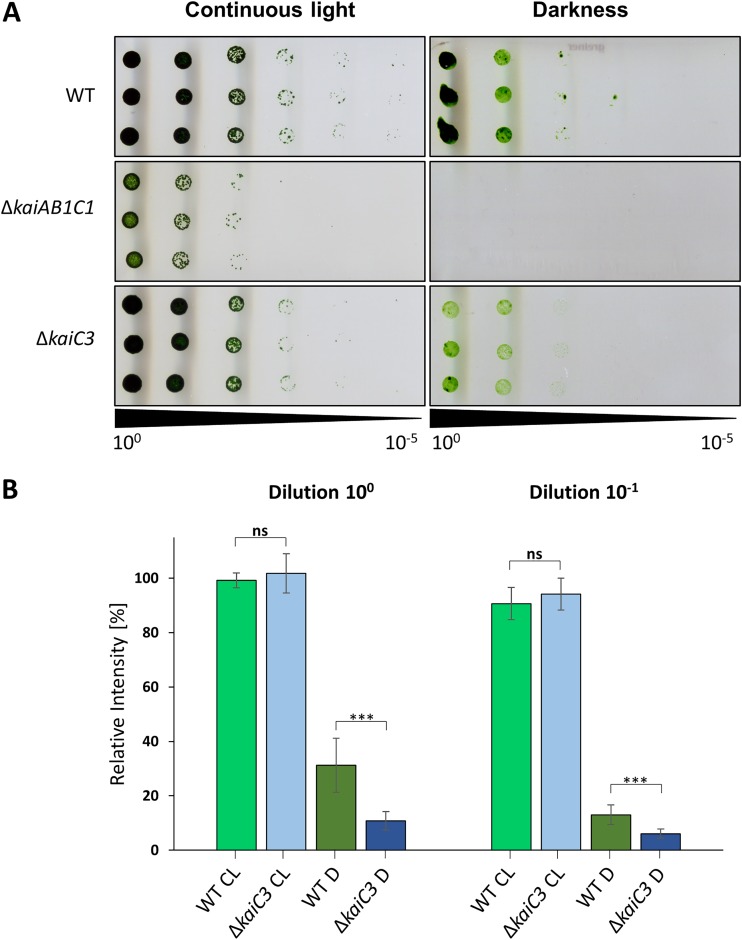
Δ*kaiC3* strain shows growth defects in complete darkness. (A) Proliferation of the *Synechocystis* wild type, the *kaiAB1C1* deletion mutant (Δ*kaiAB1C1*), and the *kaiC3* deletion mutant (Δ*kaiC3*) was tested under mixotrophic conditions in continuous light and under heterotrophic dark conditions. Strains were grown in liquid culture under constant light, and different dilutions were spotted on agar plates containing 0.2% glucose. Plates were analyzed after further incubation for 6 or 28 days of continuous light and darkness, respectively. A representative result from three independent experiments is shown. (B) Measurement of single spot densities of the *Synechocystis* wild-type and *kaiC3* deletion strains. Single spot intensities were determined with Quantity One (Bio-Rad) and normalized to the intensity of the wild-type spot of the respective control (continuous light condition). Shown are mean values with standard deviations from at least three replicates. Statistical significance was calculated by Brown-Forsythe and Welch ANOVA tests, followed by Dunnett’s T3 multiple-comparison test using GraphPad Prism 8. Significance of the mean difference is indicated as nonsignificant (*P* ≥ 0.05; ns) or extremely significant with *P* = 0.0001 to 0.001 (***). CL, continuous light; D, darkness.

As *in vitro* analyses suggested an interaction of KaiC3 with KaiB1 and KaiC1, we aimed at elucidating a putative role of KaiC3 and its cross talk with the KaiC1-based system in the cell. Clock factors are reported to be essential for cell viability in light-dark cycles (18, 22, 24). For assessing growth of mutant strains lacking *kai* genes, we used a spot plating assay (56). In this assay, the amount of cells plated on agar in serial dilutions is compared to the amount of cells that are able to form a colony. The plating efficiency then allows us to compare the sensitivity of cells to changing light conditions. Notably, this method allows only limited assertions on the growth rate of different strains. Deletion of the *kaiAB1C1* cluster of *Synechocystis* resulted in lower cell viability in light-dark cycles (39). This was even more pronounced under photomixotrophic (0.2% glucose) than photoautotrophic conditions, whereas deletion of *kaiC3* had no effect on cell viability in light-dark cycles (39). As KaiC3 homologs are also present in nonphotosynthetic bacteria (27), we were interested in the growth of the Δ*kaiC3* strain in the dark. The *Synechocystis* wild-type strain used here, in contrast to those used in previous studies (48), is able to grow in complete darkness when supplemented with glucose (22). We therefore analyzed the viability of Δ*kaiC3* cells via spot assays in constant light and in complete darkness on agar plates containing 0.2% glucose (Fig. 5A). Under photomixotrophic growth conditions with continuous illumination, wild-type and Δ*kaiC3* strains showed similar viability, whereas the viability of the Δ*kaiAB1C1* strain was reduced, reflected by a lower plating efficiency. Further, in complete darkness, the wild type displayed detectable growth, while growth of the Δ*kaiAB1C1* strain was abolished completely. Sensitivity of the Δ*kaiAB1C1* mutant to mixotrophic conditions in the light and lack of growth in the dark highlights the importance of the KaiAB1C1 oscillator for the switch between these two different metabolic modes of *Synechocystis*. The Δ*kaiC3* strain could grow in the dark but showed some impairments. The plating efficiency in serial dilutions indicates similar viability of the wild-type and Δ*kaiC3* deletion strains (Fig. 5A). However, dark growth seemed to be more impaired in the Δ*kaiC3* strain than in the wild type. For a more quantitative assessment, intensity measurements of single spots of the *Synechocystis* wild-type and *kaiC3* deletion strain were performed (Fig. 5B), validating the growth impairment of the *kaiC3* deletion strain in complete darkness. Thus, KaiC3 seems to be linked to dark adaption of *Synechocystis* cells but is not as essential as the core oscillator KaiAB1C1.

## DISCUSSION

### Characterization of enzymatic activities of KaiC3.

Previous sequence analysis suggested that the three enzymatic activities of KaiC, which are autophosphorylation, autodephosphorylation, and ATPase activity, are conserved in all cyanobacterial KaiC proteins (27). This hypothesis is supported by experiments demonstrating autophosphorylation of all *Synechocystis* KaiC proteins (31), different cyanobacterial KaiC1 and KaiC3 homologs (27, 36, 38), and even noncyanobacterial KaiC homologs from Rhodopseudomonas palustris (37), Legionella pneumophila (47), and two thermophilic *Archaea* (27). In the current paper, we provide experimental evidence that ATPase activity is conserved in KaiC3 as well. However, the level of the ATPase activity seems to vary between KaiC proteins. While ATPase activity of the KaiC from Prochlorococcus marinus MED4 was reported to have the same activity as KaiC (36), we show here that the ATPase activity of KaiC3 is lower. In contrast, ATP hydrolysis activity of the KaiC2 homolog from Rhodopseudomonas palustris and KaiC1 homolog from *Gloeocapsa* sp. strain PCC 7428 were reported to be elevated compared to the level of the standard KaiC protein (37, 38).

ATPase activity of KaiC3 further differed from that of KaiC by lacking temperature compensation (Fig. 4). Therefore, KaiC3 cannot be the core component of a true circadian oscillator. This is further supported by differences in the ATP hydrolysis rate between the phosphomimetic variants of KaiC and KaiC3. ATPase activity of the true clock protein KaiC from *S. elongatus* depends on its phosphorylation status. In the KaiC-AA variant, which mimics the dephosphorylated state of KaiC, the ATPase activity is higher than average, whereas in KaiC-DE, mimicking the phosphorylation state, it is diminished (4). In our analysis, ATP hydrolysis by Strep-KaiC3-AA was similarly increased compared to that of Strep-KaiC3. However, Strep-KaiC3-DE did not show reduced activity, as was shown for KaiC-DE, implying that the phosphorylation state of KaiC3 does not influence KaiC3’s ATPase activity.

The ATPase activity of Strep-KaiC3 was affected decisively by the addition of KaiB3 and KaiB1. This points at a putative mechanism for how KaiC3 might fine-tune the standard oscillator: binding of KaiB1 to KaiC3 will reduce the free KaiB1 concentration in the cell and thereby influence the KaiAB1C1 oscillator. Both KaiB homologs led to decreased ATPase activity of Strep-KaiC3 (Fig. 4A). One needs to take into consideration that we used 0.04 mg ml^−1^ KaiB protein for all assays. As KaiB1 was present only as a tetramer in the used fractions, this resulted in a molar concentration of 0.82 μM KaiB1 tetramers versus 3.36 μM KaiB3 monomers. *S. elongatus* KaiB and KaiB3 both exist as a monomer or tetramer in solution, and KaiB is known to bind as a monomer to KaiC (49–51). In our analysis where we separated monomeric and tetrameric KaiB3 forms, only monomeric KaiB3 showed an effect on ATP hydrolysis by KaiC3. Therefore, addition of a similar monomeric concentration (3.28 μM KaiB1 and 3.36 μM KaiB3) was important to compare the effect of KaiB1 and KaiB3. As a tetramer, KaiB subunits adopt a different unique fold, which was also observed in crystals of KaiB1 (52). Residues K57, G88, and D90, which are important for fold switching and, hence, transition between tetrameric and monomeric KaiB (51), are also conserved in KaiB3. We therefore cannot exclude that addition of a higher molar concentration of KaiB3 tetramers might affect ATPase activity of KaiC3. However, we show only *in vitro* data here. In the cell, protein concentrations as well as spatial and temporal separation of the different proteins might have an influence on these interactions. In addition, we do not take possible heteromeric interactions among the KaiB proteins into account.

### Function of KaiC3 in an extended network.

The interaction studies shown here imply a cross talk between KaiC1 and KaiC3 (Fig. 4C). KaiC1 is believed to form a standard oscillator together with KaiA_6803_ and KaiB1 (29, 31), which is supported by the KaiC1-KaiB1 interaction observed here (Fig. 3C; see also Fig. S3 in the supplemental material). We further hypothesize that KaiC3 acts in a separate noncircadian regulatory system together with KaiB3. In accordance with bioinformatic analysis, which showed a significant cooccurrence of KaiB3 and KaiC3 in *Cyanobacteria* (27), KaiB3 had a stronger effect on the ATPase activity of KaiC3 than KaiB1 (Fig. 4A). In the cell, the KaiAB1C1 and KaiB3C3 systems could work independently from each other. However, KaiC3 is able to form hetero-oligomers with KaiC1 and was shown to interact with KaiB1 as well. Therefore, interference between the KaiC3-KaiB3 system and the proteins of the standard oscillator is very likely. Further, we exclude that KaiA_6803_ is involved in the putative cross talk for the following reasons: (i) KaiA_6803_ stimulated neither ATP hydrolysis nor kinase activity of KaiC3 (this work and Wiegard et al. [31]), (ii) we were not able to show an interaction of KaiC3 and KaiA_6803_ using different approaches (this work and Wiegard et al. [31]), (iii) KaiA-interacting residues are not conserved in cyanobacterial KaiC3 homologs, and (iv) KaiC3 homologs are present in organisms that do not harbor KaiA (27).

The growth defect of the Δ*kaiAB1C1* and Δ*kaiC3* mutant strains in complete darkness suggests that the putative KaiC3-KaiB3 system and the KaiAB1C1 oscillator proposed here target similar cellular functions. The metabolic network of cyanobacteria is described as temporally partitioned with extensive effects of day-night transitions, involving shifts in ATP and reductant levels and alterations of the carbon flux (24). In *S. elongatus*, environmental signals can be fed into the main clock output system via the transcriptional regulator RpaB (53). In contrast to *S. elongatus*, *Synechocystis* is able to grow chemoheterotrophically, which adds another layer of complexity to day-night transitions and demand for further regulatory elements. The observed impaired growth of the *kaiC3* mutant in darkness supports the idea of KaiC3 and KaiB3 functioning as such additional elements to adjust the state of the main *Synechocystis* oscillator. Conversely, it is also possible that KaiC3 function is controlled by the KaiAB1C1 clock system. Köbler et al. (22) demonstrated that solely KaiC1, but not KaiC3, interacts with the main output histidine kinase SasA in the *Synechocystis* timing system. Thus, in *Synechocystis*, only the main oscillator feeds timing information into the SasA-RpaA output system to control the expression of many genes involved in dark growth (22). The output pathway for KaiC3 is unknown so far, and it is possible that the only function of KaiC3 is to modulate the function of the main oscillator in response to an unknown input factor.

## MATERIALS AND METHODS

### Cloning, expression, and purification of recombinant Kai proteins.

Genes encoding KaiB1 (open reading frame [ORF] *slr0757*) and KaiB3 (ORF *sll0486*) were amplified from genomic *Synechocystis* wild-type DNA using specific primers (see Table S1 in the supplemental material) and Phusion polymerase (New England Biolabs). After restriction digestion with BamHI and NotI, amplified fragments were inserted into pGEX-6P1 (GE Healthcare), and the resulting plasmids were used for heterologous expression. For production of recombinant KaiC as well as KaiA_6803_, KaiB1, KaiB3, and KaiC3, we used pGEX-based plasmids described in Wiegard et al. (31) (see Table S2 for a list of all plasmids used in this study). A detailed protocol of expression and purification can be found on protocols.io (https://doi.org/10.17504/protocols.io.48ggztw). Briefly, proteins were expressed as GST fusion proteins in Escherichia coli BL21(DE3) or NEB Express (New England Biolabs) and lysed in 50 mM Tris-HCl (pH 8), 150 mM NaCl, 0.5 mM EDTA, 1 mM dithiothreitol (DTT) (plus 5 mM MgCl_2_, 1 mM ATP for KaiC proteins). Purification was performed via batch affinity chromatography using glutathione-agarose 4B (Macherey and Nagel) or glutathione-Sepharose 4B (GE Healthcare) in the same buffer. Finally, the GST tag was cleaved off using PreScission protease (GE Healthcare) in 50 mM Tris-HCl (pH 8), 150 mM NaCl, 1 mM EDTA, 1 mM DTT (plus 5 mM MgCl_2_, 1 mM ATP for KaiC proteins). If homogeneity of the proteins was not sufficient, they were further purified via anion-exchange chromatography with a MonoQ or ResourceQ column (GE Healthcare Life Sciences) using 50 mM Tris-HCl (pH 8), 1 mM EDTA, 1 mM DTT (plus 5 mM MgCl_2_, 1 mM ATP for KaiC proteins), and a 0 to 1 M NaCl gradient.

To produce Strep-KaiC3 variants with amino acid substitutions, the *kaiC3* gene in the pGEX-kaiC3 vector was modified by site-directed mutagenesis using the QuikChange site-directed mutagenesis kit (Stratagene) or Q5 site-directed mutagenesis kit (New England Biolabs). Base triplets encoding S423 and T424 were changed to code for alanine or for aspartate and glutamate, resulting in *kaiC3-AA* and *kaiC3-DE* genes, respectively. To generate *kaiC3-catE1-catE2*, two subsequent site-directed mutagenesis reactions were performed to exchange nucleotides encoding E67 and E68 as well as nucleotides encoding E310 and E311 with bases encoding glutamine (all primers used for mutagenesis are listed in Table S1). Afterwards, *kaiC3* WT and modified genes were amplified with KOD-Plus-Neo polymerase (Toyobo) using pASK-kaiC3 primers (Table S1). Amplicons were digested with SacII and HindIII and ligated into the respective restriction sites of pASK-IBA5plus (IBA Life Sciences). For purification of recombinant Strep-KaiC, the pASK-IBA-5plus-based vector described in Oyama et al. (14) was used. Strep-KaiC3 proteins were expressed in E. coli Rosetta gamiB(DE3) or Rosetta gami2(DE3) cells (Novagen). Expression of Strep-KaiC was carried out in E. coli DH5α. Cells were cultured in LB medium containing 100 μg ml^−1^ ampicillin with vigorous agitation at 37°C. At an optical density at 600 nm (OD_600_) of 0.33 to 0.68, protein expression was induced with 200 ng ml^−1^ anhydrotetracycline and the strains were further incubated with the following parameters: Strep-KaiC, 7 h at 37°C; Strep-KaiC3-WT, 5 h at 35°C or 3.5 h at 37°C; Strep-KaiC3-AA and Strep-KaiC3-catE1-catE2-, 18°C overnight; Strep-KaiC3-DE, 25°C overnight. Cells were harvested and lysed by sonication in ice-cold buffer W (20 mM Tris-HCl [pH 8], 150 mM NaCl, 5 mM MgCl_2_, 1 mM ATP [plus 2 mM DTT for Strep-KaiC3 proteins]) including protease inhibitors (protease inhibitor cocktail; Roche or Nacalai). Soluble proteins were loaded on self-prepared columns packed with Strep-Tactin XT Superflow or Strep-Tactin Sepharose (IBA Life Sciences) and purified under gravity flow. After washing with buffer W, Strep-KaiC proteins were eluted with ice-cold buffer W plus 50 mM D(+)biotin (for Strep-Tactin XT Superflow) or ice-cold buffer W plus 2.5 mM desthiobiotin (for Strep-Tactin Superflow). See https://doi.org/10.17504/protocols.io.meac3ae for a detailed protocol.

All proteins used for ATPase activity measurements were further purified via size exclusion chromatography. Strep-KaiC3 and Strep-KaiC proteins were applied on a Sephacryl S300 HR HiPrep 16/60 Sephacryl column (GE Healthcare) and separated in 20 or 50 mM Tris-HCl (pH 8.0), 150 mM NaCl, 2 mM DTT, 1 mM ATP, and 5 mM MgCl_2_. For separation of KaiB1 and KaiB3, a Sephacryl S200 HR HiPrep 16/60 column (GE Healthcare) or Superdex 200 Increase 10/30 GL column (GE Healthcare) and 20 or 50 mM Tris-HCl (pH 8.0), 150 mM NaCl, 2 mM DTT as running buffer were used. KaiA_6803_ was purified on a Superdex 200 Increase 10/30 GL column (GE Healthcare) in 20 mM Tris-HCl (pH 8.0), 150 mM NaCl, 2 mM DTT. See https://doi.org/10.17504/protocols.io.mdtc26n for further details.

### ATPase activity.

For ATPase measurements, KaiC proteins fused to an N-terminal Strep-tag were used, and all Kai proteins were purified via size exclusion chromatography (described above). Strep-KaiC3 or Strep-KaiC3 variants at a concentration of 3.45 μM protein were incubated in ATPase buffer (20 mM Tris-HCl [pH 8], 150 mM NaCl, 1 mM ATP, 5 mM MgCl_2_) at 25°C, 30°C, or 35°C for 24 h. To analyze the influence of KaiA_6803_ and KaiB proteins on KaiC3 ATPase activity, we mixed 0.2 mg ml^−1^ KaiC3 with 0.04 mg ml^−1^ KaiA_6803_ or 0.04 mg ml^−1^ KaiB and incubated the mixtures for 24 h at 30°C. Monomeric and oligomeric KaiB3 were analyzed separately. To monitor ADP production, every 3, 4, or 6 h, 2 μl of the reaction mixture was applied on a Shim-Pack-VP-ODS column (Shimadzu) and separated using 100 mM phosphoric acid, 150 mM triethylamine, 1% acetonitrile as running buffer. ADP production per monomer KaiC and 24 h was calculated using a calibration curve. A detailed protocol can be found on protocols.io (https://doi.org/10.17504/protocols.io.mebc3an). The *Q*_10_ value was calculated from ATPase measurements at 25°C and 35°C, using the formula $Q_{10}={\left(\frac{R_{2}}{R_{1}}\right)}^{10{}^{\circ}\text{C}/(T_{2}-T_{1})}$.

### ATP synthase activity.

To investigate dephosphorylation via ATP synthesis, we used KaiC proteins that were expressed as GST fusion proteins and subsequently cleaved off their GST tag. KaiC (3 μM) in ATP synthesis buffer (20 mM Tris-HCl[pH 8], 150 mM NaCl, 0.5 mM EDTA, 5 mM MgCl_2_, 0.5 mM ATP) was mixed with 0.8 μCi ml^−1^ [α-^32^P]ADP and stored at –20°C or incubated for 2 h at 30°C. As a control, the same experiment was performed in the presence of 0.5 mM ADP. After 20-fold dilution, nucleotides in a 0.5-μl reaction mixture were separated via thin-layer chromatography using TLC PEI cellulose F plates (Merck Millipore) and 1 M LiCl as the solvent. [α-^32^P]ADP and [γ-^32^P]ATP were separated in parallel to identify the signals corresponding to ADP and ATP, respectively. Dried plates were subjected to autoradiography, and signals were analyzed using a Personal Molecular Imager FX system (Bio-Rad) and ImageLab software (Bio-Rad). For each reaction mixture, the relative intensity of [α-^32^P]ATP was calculated as a percentage of all signals in the corresponding lane. Because [α-^32^P]ATP was already synthesized during mixing of the samples, the relative ATP intensity measured in the –20°C sample containing 0.5 mM ADP was subtracted for normalization. The principle of this method is based on Egli et al. (12). A detailed protocol is available on protocols.io (https://doi.org/10.17504/protocols.io.48qgzvw).

### Yeast two-hybrid assays.

For yeast two-hybrid assays, vectors containing the GAL4 activation domain (AD) and the GAL4 DNA-binding domain (BD) were used. Genes of interest were amplified from *Synechocystis* wild-type genomic DNA with Phusion polymerase (NEB) according to the manufacturer’s guidelines. Indicated restriction sites were introduced via the oligonucleotides listed in Table S1. Vectors and PCR fragments were cut with the respective restriction enzymes (Thermo Fisher Scientific), and the gene of interest was ligated into the vector, leading to a fusion protein with an AD or BD tag either at the N or C terminus. All constructed plasmids are listed in Table S2. Transformation of yeast cells was performed according to the manufacturer’s guidelines using the Frozen-EZ yeast transformation II kit (Zymo Research), and cells were selected on complete supplement mixture (CSM) lacking leucine and tryptophan (−Leu −Trp) dropout medium (MP Biochemicals) at 30°C for 3 to 4 days. Y190 (Clontech) cells were used for measuring β‑galactosidase activity. Formed colonies were spotted on a second plate (CSM −Leu −Trp) and incubated for 2 days. A colony-lift filter assay then was performed as described by Breeden and Nasmyth (54). A detailed protocol can be found on protocols.io (https://doi.org/10.17504/protocols.io.v7ve9n6).

### Bacterial strains and growth conditions.

Wild type *Synechocystis* sp. strain PCC 6803 (PCC-M, resequenced [45]) and the *kaiC3* deletion mutant (39) were cultured photoautotrophically in BG11 medium (55) supplemented with 10 mM *N*-tris(hydroxymethyl)methyl-2-aminoethanesulfonic acid buffer (pH 8) under constant illumination with 50 μmol photons m^−2^ s^−1^ of white light (Philips TLD Super 80/840) at 30°C. Cells were grown either in Erlenmeyer flasks with constant shaking (140 rpm) or on plates (0.75% Bacto agar; Difco) supplemented with 0.3% thiosulfate. Detailed recipes can be found on protocols.io (https://doi.org/10.17504/protocols.io.wj5fcq6).

### Spot assays.

Experiments were performed as previously described (56). Strains were propagated mixotrophically on BG11 agar plates with the addition of 0.2% (wt/vol) glucose. Dilution series of cell cultures started with OD_750_ of 0.2 and OD_750_ of 0.4, followed by incubation of the plates for 6 or 28 days under constant light conditions and in complete darkness, respectively. Plates were scanned and single-spot intensities were quantified using Quantity One (Bio-Rad). Measured intensities were normalized to the intensity of the wild-type spot of the respective control grown under continuous light.

## Supplementary Material

Supplemental file 1

## References

[1] Ditty JL, Williams SB, Golden SS 2003 A cyanobacterial circadian timing mechanism. Annu Rev Genet 37:513–543. doi:10.1146/annurev.genet.37.110801.142716. 14616072

[2] Nakajima M, Imai K, Ito H, Nishiwaki T, Murayama Y, Iwasaki H, Oyama T, Kondo T 2005 Reconstitution of circadian oscillation of cyanobacterial KaiC phosphorylation *in vitro*. Science 308:414–415. doi:10.1126/science.1108451. 15831759

[3] Iwasaki H, Nishiwaki T, Kitayama Y, Nakajima M, Kondo T 2002 KaiA-stimulated KaiC phosphorylation in circadian timing loops in cyanobacteria. Proc Natl Acad Sci U S A 99:15788–15793. doi:10.1073/pnas.222467299. 12391300 PMC137794

[4] Terauchi K, Kitayama Y, Nishiwaki T, Miwa K, Murayama Y, Oyama T, Kondo T 2007 ATPase activity of KaiC determines the basic timing for circadian clock of cyanobacteria. Proc Natl Acad Sci U S A 104:16377–16381. doi:10.1073/pnas.0706292104. 17901204 PMC2042214

[5] Kitayama Y, Iwasaki H, Nishiwaki T, Kondo T 2003 KaiB functions as an attenuator of KaiC phosphorylation in the cyanobacterial circadian clock system. EMBO J 22:2127–2134. doi:10.1093/emboj/cdg212. 12727879 PMC156084

[6] Hayashi F, Suzuki H, Iwase R, Uzumaki T, Miyake A, Shen JR, Imada K, Furukawa Y, Yonekura K, Namba K, Ishiura M 2003 ATP-induced hexameric ring structure of the cyanobacterial circadian clock protein KaiC. Genes Cells 8:287–296. doi:10.1046/j.1365-2443.2003.00633.x. 12622725

[7] Mori T, Saveliev SV, Xu Y, Stafford WF, Cox MM, Inman RB, Johnson CH 2002 Circadian clock protein KaiC forms ATP-dependent hexameric rings and binds DNA. Proc Natl Acad Sci U S A 99:17203–17208. doi:10.1073/pnas.262578499. 12477935 PMC139293

[8] Pattanayek R, Wang J, Mori T, Xu Y, Johnson CH, Egli M 2004 Visualizing a circadian clock protein: crystal structure of KaiC and functional insights. Mol Cell 15:375–388. doi:10.1016/j.molcel.2004.07.013. 15304218

[9] Nishiwaki T, Iwasaki H, Ishiura M, Kondo T 2000 Nucleotide binding and autophosphorylation of the clock protein KaiC as a circadian timing process of cyanobacteria. Proc Natl Acad Sci U S A 97:495–499. doi:10.1073/pnas.97.1.495. 10618446 PMC26691

[10] Murakami R, Miyake A, Iwase R, Hayashi F, Uzumaki T, Ishiura M 2008 ATPase activity and its temperature compensation of the cyanobacterial clock protein KaiC. Genes Cells 13:387–395. doi:10.1111/j.1365-2443.2008.01174.x. 18363969

[11] Nishiwaki T, Kondo T 2012 Circadian autodephosphorylation of cyanobacterial clock protein KaiC occurs via formation of ATP as intermediate. J Biol Chem 287:18030–18035. doi:10.1074/jbc.M112.350660. 22493509 PMC3365771

[12] Egli M, Mori T, Pattanayek R, Xu Y, Qin X, Johnson CH 2012 Dephosphorylation of the core clock protein KaiC in the cyanobacterial KaiABC circadian oscillator proceeds via an ATP synthase mechanism. Biochemistry 51:1547–1558. doi:10.1021/bi201525n. 22304631 PMC3293397

[13] Phong C, Markson JS, Wilhoite CM, Rust MJ 2013 Robust and tunable circadian rhythms from differentially sensitive catalytic domains. Proc Natl Acad Sci U S A 110:1124–1129. doi:10.1073/pnas.1212113110. 23277568 PMC3549141

[14] Oyama K, Azai C, Nakamura K, Tanaka S, Terauchi K 2016 Conversion between two conformational states of KaiC is induced by ATP hydrolysis as a trigger for cyanobacterial circadian oscillation. Sci Rep 6:32443. doi:10.1038/srep32443. 27580682 PMC5007536

[15] Shultzaberger RK, Boyd JS, Diamond S, Greenspan RJ, Golden SS 2015 Giving time purpose: the Synechococcus elongatus clock in a broader network context. Annu Rev Genet 49:485–505. doi:10.1146/annurev-genet-111212-133227. 26442846 PMC4769874

[16] Dong G, Yang Q, Wang Q, Kim YI, Wood TL, Osteryoung KW, van Oudenaarden A, Golden SS 2010 Elevated ATPase activity of KaiC applies a circadian checkpoint on cell division in *Synechococcus elongatus*. Cell 140:529–539. doi:10.1016/j.cell.2009.12.042. 20178745 PMC3031423

[17] Swan JA, Golden SS, LiWang A, Partch CL 2018 Structure, function, and mechanism of the core circadian clock in cyanobacteria. J Biol Chem 293:5026–5034. doi:10.1074/jbc.TM117.001433. 29440392 PMC5892564

[18] Diamond S, Jun D, Rubin BE, Golden SS 2015 The circadian oscillator in Synechococcus elongatus controls metabolite partitioning during diurnal growth. Proc Natl Acad Sci U S A 112:E1916–E1925. doi:10.1073/pnas.1504576112. 25825710 PMC4403147

[19] Welkie DG, Rubin BE, Diamond S, Hood RD, Savage DF, Golden SS 2019 A hard day’s night: cyanobacteria in Diel cycles. Trends Microbiol 27:231–242. doi:10.1016/j.tim.2018.11.002. 30527541 PMC6377297

[20] Pattanayak G, Rust MJ 2014 The cyanobacterial clock and metabolism. Curr Opin Microbiol 18:90–95. doi:10.1016/j.mib.2014.02.010. 24667330 PMC4068238

[21] Woelfle MA, Ouyang Y, Phanvijhitsiri K, Johnson CH 2004 The adaptive value of circadian clocks: an experimental assessment in cyanobacteria. Curr Biol 14:1481–1486. doi:10.1016/j.cub.2004.08.023. 15324665

[22] Köbler C, Schultz SJ, Kopp D, Voigt K, Wilde A 2018 The role of the Synechocystis sp. PCC 6803 homolog of the circadian clock output regulator RpaA in day-night transitions. Mol Microbiol 110:847–861. doi:10.1111/mmi.14129. 30216574

[23] Ungerer J, Wendt KE, Hendry JI, Maranas CD, Pakrasi HB 2018 Comparative genomics reveals the molecular determinants of rapid growth of the cyanobacterium Synechococcus elongatus UTEX. Proc Natl Acad Sci U S A 115:E11761–E11770. doi:10.1073/pnas.1814912115. 30409802 PMC6294925

[24] Welkie DG, Rubin BE, Chang YG, Diamond S, Rifkin SA, LiWang A, Golden SS 2018 Genome-wide fitness assessment during diurnal growth reveals an expanded role of the cyanobacterial circadian clock protein KaiA. Proc Natl Acad Sci U S A 115:E7174–E7183. doi:10.1073/pnas.1802940115. 29991601 PMC6064986

[25] Diamond S, Rubin BE, Shultzaberger RK, Chen Y, Barber CD, Golden SS 2017 Redox crisis underlies conditional light-dark lethality in cyanobacterial mutants that lack the circadian regulator, RpaA. Proc Natl Acad Sci U S A 114:E580–E589. doi:10.1073/pnas.1613078114. 28074036 PMC5278464

[26] Shih PM, Wu D, Latifi A, Axen SD, Fewer DP, Talla E, Calteau A, Cai F, Tandeau de Marsac N, Rippka R, Herdman M, Sivonen K, Coursin T, Laurent T, Goodwin L, Nolan M, Davenport KW, Han CS, Rubin EM, Eisen JA, Woyke T, Gugger M, Kerfeld CA 2013 Improving the coverage of the cyanobacterial phylum using diversity-driven genome sequencing. Proc Natl Acad Sci U S A 110:1053–1058. doi:10.1073/pnas.1217107110. 23277585 PMC3549136

[27] Schmelling NM, Lehmann R, Chaudhury P, Beck C, Albers S-V, Axmann IMM, Wiegard A 2017 Minimal tool set for a prokaryotic circadian clock. bioRxiv https://www.biorxiv.org/content/10.1101/075291v2. 10.1186/s12862-017-0999-7 PMC5520375 28732467

[28] Axmann IM, Hertel S, Wiegard A, Dorrich AK, Wilde A 2014 Diversity of KaiC-based timing systems in marine Cyanobacteria. Mar Genomics 14:3–16. doi:10.1016/j.margen.2013.12.006. 24388874

[29] Aoki S, Onai K 2009 Circadian clocks of *Synechocystis* sp. strain PCC 6803, *Thermosynechococcus elongatus*, *Prochlorococcus* spp., *Trichodesmium* spp. and other species, p 259–282. *In* Ditty JL, Mackey SR, Johnson CH (ed), Bacterial circadian programs. Springer, Berlin, Germany.

[30] Dvornyk V, Vinogradova O, Nevo E 2003 Origin and evolution of circadian clock genes in prokaryotes. Proc Natl Acad Sci U S A 100:2495–2500. doi:10.1073/pnas.0130099100. 12604787 PMC151369

[31] Wiegard A, Dörrich AK, Deinzer HT, Beck C, Wilde A, Holtzendorff J, Axmann IM 2013 Biochemical analysis of three putative KaiC clock proteins from *Synechocystis* sp. PCC 6803 suggests their functional divergence. Microbiology 159:948–958. doi:10.1099/mic.0.065425-0. 23449916

[32] Johnson CH, Egli M 2014 Metabolic compensation and circadian resilience in prokaryotic cyanobacteria. Annu Rev Biochem 83:221–247. doi:10.1146/annurev-biochem-060713-035632. 24905782 PMC4259047

[33] Holtzendorff J, Partensky F, Mella D, Lennon JF, Hess WR, Garczarek L 2008 Genome streamlining results in loss of robustness of the circadian clock in the marine cyanobacterium *Prochlorococcus marinus* PCC 9511. J Biol Rhythms 23:187–199. doi:10.1177/0748730408316040. 18487411

[34] Kanesaki Y, Shiwa Y, Tajima N, Suzuki M, Watanabe S, Sato N, Ikeuchi M, Yoshikawa H 2012 Identification of substrain-specific mutations by massively parallel whole-genome resequencing of Synechocystis sp. PCC 6803. DNA Res 19:67–79. doi:10.1093/dnares/dsr042. 22193367 PMC3276265

[35] Dvornyk V, Knudsen B 2005 Functional divergence of the circadian clock proteins in prokaryotes. Genetica 124:247–254. doi:10.1007/s10709-005-3146-0. 16134337

[36] Axmann IM, Dühring U, Seeliger L, Arnold A, Vanselow JT, Kramer A, Wilde A 2009 Biochemical evidence for a timing mechanism in *Prochlorococcus*. J Bacteriol 191:5342–5347. doi:10.1128/JB.00419-09. 19502405 PMC2725622

[37] Ma P, Mori T, Zhao C, Thiel T, Johnson CH 2016 Evolution of KaiC-dependent timekeepers: a proto-circadian timing mechanism confers adaptive fitness in the purple bacterium Rhodopseudomonas palustris. PLoS Genet 12:e1005922. doi:10.1371/journal.pgen.1005922. 26982486 PMC4794148

[38] Mukaiyama A, Ouyang D, Furuike Y, Akiyama S 2019 KaiC from a cyanobacterium Gloeocapsa sp. PCC 7428 retains functional and structural properties required as the core of circadian clock system. Int J Biol Macromol 131:67–73. doi:10.1016/j.ijbiomac.2019.03.051. 30857964

[39] Dörrich AK, Mitschke J, Siadat O, Wilde A 2014 Deletion of the Synechocystis sp. PCC 6803 kaiAB1C1 gene cluster causes impaired cell growth under light-dark conditions. Microbiology 160:2538–2550. doi:10.1099/mic.0.081695-0. 25139948

[40] Kucho K-I, Okamoto K, Tsuchiya Y, Nomura S, Nango M, Kanehisa M, Ishiura M 2005 Global analysis of circadian expression in the cyanobacterium *Synechocystis* sp. strain PCC 6803. J Bacteriol 187:2190–2199. doi:10.1128/JB.187.6.2190-2199.2005. 15743968 PMC1064041

[41] Beck C, Hertel S, Rediger A, Lehmann R, Wiegard A, Kolsch A, Heilmann B, Georg J, Hess WR, Axmann IM 2014 Daily expression pattern of protein-coding genes and small noncoding RNAs in Synechocystis sp. strain PCC 6803. Appl Environ Microbiol 80:5195–5206. doi:10.1128/AEM.01086-14. 24928881 PMC4136122

[42] van Alphen P, Hellingwerf KJ 2015 Sustained circadian rhythms in continuous light in Synechocystis sp. PCC6803 growing in a well-controlled photobioreactor. PLoS One 10:e0127715. doi:10.1371/journal.pone.0127715. 26030367 PMC4452363

[43] Saha R, Liu D, Hoynes-O’Connor A, Liberton M, Yu J, Bhattacharyya-Pakrasi M, Balassy A, Zhang F, Moon TS, Maranas CD, Pakrasi HB 2016 Diurnal regulation of cellular processes in the cyanobacterium Synechocystis sp. strain PCC 6803: insights from transcriptomic, fluxomic, and physiological analyses. mBio 7:e00464-16. doi:10.1128/mBio.00464-16. 27143387 PMC4959675

[44] Zavřel T, Očenášová P, Červený J 2017 Phenotypic characterization of Synechocystis sp. PCC 6803 substrains reveals differences in sensitivity to abiotic stress. PLoS One 12:e0189130. doi:10.1371/journal.pone.0189130. 29216280 PMC5720811

[45] Trautmann D, Voss B, Wilde A, Al-Babili S, Hess WR 2012 Microevolution in cyanobacteria: re-sequencing a motile substrain of Synechocystis sp. PCC 6803. DNA Res 19:435–448. doi:10.1093/dnares/dss024. 23069868 PMC3514855

[46] Wiegard A, Köbler C, Oyama K, Dörrich AK, Azai C, Terauchi K, Wilde A, Axmann IM 2019 Synechocystis KaiC3 displays temperature and KaiB dependent ATPase activity and is important for viability in darkness. bioRxiv doi:10.1101/700500. PMC6989803 31767776

[47] Loza-Correa M, Sahr T, Rolando M, Daniels C, Petit P, Skarina T, Gomez Valero L, Dervins-Ravault D, Honoré N, Savchenko A, Buchrieser C 2014 The Legionella pneumophila kai operon is implicated in stress response and confers fitness in competitive environments. Environ Microbiol 16:359–381. doi:10.1111/1462-2920.12223. 23957615 PMC4113418

[48] Anderson SL, McIntosh L 1991 Light-activated heterotrophic growth of the cyanobacterium Synechocystis sp. strain PCC 6803: a blue-light-requiring process. J Bacteriol 173:2761–2767. doi:10.1128/jb.173.9.2761-2767.1991. 1902208 PMC207855

[49] Snijder J, Schuller JM, Wiegard A, Lossl P, Schmelling N, Axmann IM, Plitzko JM, Forster F, Heck AJ 2017 Structures of the cyanobacterial circadian oscillator frozen in a fully assembled state. Science 355:1181–1184. doi:10.1126/science.aag3218. 28302852

[50] Tseng R, Goularte NF, Chavan A, Luu J, Cohen SE, Chang YG, Heisler J, Li S, Michael AK, Tripathi S, Golden SS, LiWang A, Partch CL 2017 Structural basis of the day-night transition in a bacterial circadian clock. Science 355:1174–1180. doi:10.1126/science.aag2516. 28302851 PMC5441561

[51] Chang YG, Cohen SE, Phong C, Myers WK, Kim YI, Tseng R, Lin J, Zhang L, Boyd JS, Lee Y, Kang S, Lee D, Li S, Britt RD, Rust MJ, Golden SS, LiWang A 2015 Circadian rhythms. A protein fold switch joins the circadian oscillator to clock output in cyanobacteria. Science 349:324–328. doi:10.1126/science.1260031. 26113641 PMC4506712

[52] Hitomi K, Oyama T, Han S, Arvai AS, Getzoff ED 2005 Tetrameric architecture of the circadian clock protein KaiB. A novel interface for intermolecular interactions and its impact on the circadian rhythm. J Biol Chem 280:19127–19135. doi:10.1074/jbc.M411284200. 15716274

[53] Espinosa J, Boyd JS, Cantos R, Salinas P, Golden SS, Contreras A 2015 Cross-talk and regulatory interactions between the essential response regulator RpaB and cyanobacterial circadian clock output. Proc Natl Acad Sci U S A 112:2198–2203. doi:10.1073/pnas.1424632112. 25653337 PMC4343135

[54] Breeden L, Nasmyth K 1985 Regulation of the yeast HO gene. Cold Spring Harbor Symp Quant Biol 50:643–650. doi:10.1101/sqb.1985.050.01.078. 3938367

[55] Rippka R, Deruelles J, Waterbury JB, Herdman M, Stanier RY 1979 Generic assignments, strain histories and properties of pure cultures of cyanobacteria. J Gen Microbiol 111:1–61. doi:10.1099/00221287-111-1-1.

[56] Dörrich AK, Wilde A 2015 Spot assays for viability analysis of cyanobacteria. Bio-protocol 5:e1574. doi:10.21769/BioProtoc.1574.

