# Supplementary material for: Synechocystis KaiC3 Displays Temperature- and KaiB-Dependent ATPase Activity and Is Important for Growth in Darkness
Source: J Bacteriol. 2020 Jan 29;202(4):e00478-19. doi: 10.1128/JB.00478-19 (PMC6989803; doi:10.1128/JB.00478-19)
Supplement: Supplemental file 1 [file JB.00478-19-s0001.pdf]

Supplemental material

Title: *Synechocystis* KaiC3 displays temperature and KaiB dependent ATPase activity and is important for growth in darkness

Running title: Biochemical characterization of *Synechocystis* KaiC3

Authors: Anika Wiegard[1,*,#], Christin Köbler[2], Katsuaki Oyama[3,‡], Anja K. Dörrich[4], Chihiro Azai[3,5], Kazuki Terauchi[3,5,#], Annegret Wilde[2], Ilka M. Axmann[1]

Author affiliations:

[1] Institute for Synthetic Microbiology, Cluster of Excellence on Plant Sciences (CEPLAS), Heinrich Heine University Duesseldorf, Duesseldorf, Germany

[2] Institute of Biology III, Faculty of Biology, University of Freiburg, Freiburg, Germany

[3] Graduate School of Life Sciences, Ritsumeikan University, Kusatsu, Shiga, Japan

[4] Institute for Microbiology and Molecular Biology, Justus-Liebig University, Giessen, Germany

[5] College of Life Sciences, Ritsumeikan University, Kusatsu, Shiga, Japan

* present address: Department of Cell and Molecular Biology, Karolinska Institutet, Stockholm, Sweden

‡ present address: Graduate School of Medicine, Kobe University, Kobe, Hyougo, Japan

# Correspondence and requests for materials should be addressed to A. Wiegard (email: anika.wiegard@ki.se) or K. Terauchi (email: terauchi@fc.ritsumei.ac.jp)

Supplementary methods

Pull down analysis of KaiB and KaiC proteins

GST-KaiB1 and GST-KaiB3 proteins were expressed in *E. coli* BL21 using the pGEX-6P1 vector and extracted as described in the main text. Protein concentration of the generated whole cell lysate was determined using the Bradford method (Bradford MM. 1976. Anal Biochem 72:248-54). For immobilization of each KaiB protein, a volume corresponding to 40 mg whole protein content was incubated with 100 µl glutathione sepharose 4B (GE Healthcare) for 20 min at room temperature. The resin was thoroughly washed four times with extraction buffer [50 mM Tris/HCl (pH8), 150 mM NaCl, 0.5 mM EDTA, 1 mM DTT (+ 5 mM $MgCl_2$, 1 mM ATP for KaiC proteins)] and subsequently incubated with *Synechocystis* wild type whole cell lysate, which had been generated from 200 ml cell culture grown to an OD_{750nm} of ~0.8. Briefly, cells were lysed in cold thylakoid buffer [50 mM Hepes/NaOH (pH7), 5 mM $MgCl_2$, 25 mM $CaCl_2$, 10% Glycerol] using glass beads (mixture of 0.1–0.11 mm and 0.25-0.5 mm size) in a bead beater (Reetsch) at 4 °C. 1 ml of the lysate was used for incubation with glutathione sepharose-bound GST-KaiB1 and GST-KaiB3 proteins to co-precipitate Kai interaction partners. Afterwards, the resin material was thoroughly washed with extraction buffer, mixed with SDS loading dye and incubated at 50 °C for 30 min before being subjected to SDS-PAGE and Western Blot analysis.

Expression and purification of 3xFLAG-tagged KaiC1 and KaiC3 proteins was performed as described earlier (Wiegard A, Dörrich AK, Deinzer HT, Beck C, Wilde A, Holtzendorff J, Axmann IM. 2013. Microbiology 159:948-958) from *Synechocystis*. After binding of 3xFLAG-KaiC1/3 to ANTI-FLAG M2 affinity gel (Sigma Aldrich) over night at 4 °C, the resin was thoroughly washed with cold FLAG buffer containing 0.03 % β-DM, followed by one washing step without the

addition of β-DM. For pull down of KaiB proteins, 150 µl resin-bound 3x-FLAG-KaiC1 and 3xFLAG-KaiC3 were incubated with 100 µl of *E. coli* BL21 whole cell lysate (adjusted to a protein content of 10 µg/µl) containing GST-KaiB1 and GST-KaiB3, respectively. After removing unbound proteins by washing with FLAG buffer, the samples were subjected to SDS-PAGE and Western Blot analysis.

Antibodies

Antibodies against KaiB1 and KaiB3 were produced as whole-rabbit-IgG fraction by Pineda Antikörper Services using synthetic peptides coupled to keyhole limpet haemocyanin as antigens. For the synthetic peptides, epitopes were completed N- and C-terminally to 15 AA. The peptide sequences were CIDVLK**NPQLAEEDKIL**AT (bold epitope corresponds to AA 50-59 in KaiB1) and CLDIVPEG**LQVRL**PED (bold epitope corresponds to AA 95-98 in KaiB3), respectively. For detection of KaiC1 and KaiC3 the specific peptide derived antibodies described in Wiegard *et al.* were used (Wiegard A, Dörrich AK, Deinzer HT, Beck C, Wilde A, Holtzendorff J, Axmann IM. 2013. Microbiology 159:948-958). HemA antibody was produced by Pineda Antikörper Services using the recombinant protein and has been described in Sobotka *et al.* (Sobotka R, Tichy M, Wilde A, Hunter CN. 2011. Plant Physiol 155:1735-47). The antibody against AtpB was kindly provided by K.-D. Irrgang (Technical University Berlin, Germany).

Oligonucleotide Name	Sequence (5' – 3')	Purpose#
Construction of yeast two-hybrid expression vectors		
KaiC3-AD-fw	taGGATCCATGATCGACCAAGAGACAG	Y2H
KaiC3-AD-rev	gcTCTAGATATTTTCTCATCGAATAAACCG	Y2H
AD-KaiC3-fw	taGGATCCATATCGACCAAGAGACAGATG	Y2H
AD-KaiC3-rev	atCTCGAGTATTTTCTCATCGAATAAACCG	Y2H
BD-KaiC3-fw	taGGATCCAATCGACCAAGAGACAGATG	Y2H
BD-KaiC3-rev	gcACTAGTTATTTTCTCATCGAATAAACCG	Y2H
AD-KaiA-fw	taGGATCCGTCAGTCTCCCCTCTCCC	Y2H
AD-KaiA-rev	taCTCGAGATCCGTCTGATAATATACATCAAAG	Y2H
BD-KaiA-fw	taGGATCCGCAGTCTCCCCTCTCCC	Y2H
BD-KaiA-rev	gcACTAGTATCCGTCTGATAATATACATCAAAG	Y2H
KaiB3-AD-fw	taGGATCCATGGATATGAATAGGATTGTGTTAAG	Y2H
KaiB3-AD-rev	gcTCTAGAATCCTCCGGCAAACG	Y2H
AD-KaiB3-fw	taGGATCCCTGATATGAATAGGATTGTGTTAAGAC	Y2H
AD-KaiB3-rev	taCTCGAGATCCTCCGGCAAACG	Y2H
BD-KaiC1-fw	aaGGATCCGAACTTACCGATTGTTAACGAAC	Y2H
BD-KaiC1-rev	gcACTAGTCTCAGCGGTCTTGTCC	Y2H
KaiB1-AD-fw	taGGATCCATGAGCCCCTTTAAAAAAAC	Y2H
KaiB1-AD-rev	gcACTAGTTTGGTCTTCTGCTTCCC	Y2H
AD-KaiB1-fw	taGGATCCGGAGCCCCTTTAAAAAAACTTAC	Y2H
AD-KaiB1-rev	ttCTCGAGTTGGTCTTCTGCTTCCC	Y2H
KaiB1-BD-rev	ttGTCGACTTTTGGTCTTCTGCTTCCC	Y2H
BD-KaiB1-fw	taGGATCCGAGCCCCTTTAAAAAAACTTAC	Y2H
Construction of vectors for heterologous expression in *E. coli*		
kaiB1-fw	CCGTGGATCCAGCCCCTTTAAAAAAACT	E
kaiB1-rev	CCTGGCGGCCGCTTTTCTATTGGTC	E
kaiB3-fw	GTTATCAGGATCCGATATGAATAGGATTGTG	E
kaiB3-rev	CTAGGGGCGGCCGCTTAATCCTCC	E
MU-kaiC3-AA-fw	GGGGAGCTTCCATTACCGATGCCCATATTGCAGCAATTACCGATTCG	MU
MU-kaiC3-AA-rev	CGAATCGGTAATTGCTGCAATATGGGCATCGGTAATGGAAGCTCCCC	MU
MU-kaiC3-DE-fw	GGGGAGCTTCCATTACCGATGCCCATATTGACGAAATTACCGATTCG	MU
MU-kaiC3-DE-rev	CGAATCGGTAATTTCGTCAATATGGGCATCGGTAATGGAAGCTCCCC	MU
MU-kaiC3-E67QE68Q-fw	TGTCACTTTTCAGCAACCCCCCAAGG	MU
MU-kaiC3-E67QE68Q-fw	AAAACACCGTTTTCCCCC	MU
MU-kaiC3-E310QE311Q-fw	TTTTGCCTTTCAGCAAAGTCGAGAACAATTAATTC	MU
MU-kaiC3-E310QE311Q-fw	ACTAAACAGCGCTCTCCATTG	MU
pASK-kaiC3-fw	ATCCGCGGTCATCGACCAAGAGACAGATG	E
pASK-kaiC3-rev	TCGCAAGCTTTATATTTTCTCATCGAATAAACC	E

Table S1. Oligonucleotides used in this study. Restriction sites are underlined. # Y2H, expression in yeast cells; E, expression in *E. coli* cells; MU, mutagenesis

Plasmid Name	Description	Reference
pCGADT7ah	Expression of fusion proteins with a C-terminal *GAL4*$_{(768–881)}$ AD-tag in yeast cells, *LEU2*, HA epitope tag	(1)
pGADT7ah	Expression of fusion proteins with an N-terminal *GAL4*$_{(768–881)}$ AD-tag in yeast cells, *LEU2*, HA epitope tag	(2)
pD153	Expression of fusion proteins with a C-terminal GAL4$_{(1–147)}$ DNA-BD-tag in yeast cells, *TRP1*, c-Myc epitope tag	(3)
pGBKT7	Expression of fusion proteins with an N-terminal GAL4$_{(1–147)}$ DNA-BD-tag in yeast cells, *TRP1*, c-Myc epitope tag	Clontech, Germany
pCGAD-*kaiC3*-AD	Expression of KaiC3$_{6803}$ with a C-terminal *GAL4*$_{(768–881)}$ AD-tag in yeast cells, *LEU2*, HA epitope tag	This study
pGAD-AD-*kaiC3*	Expression of KaiC3$_{6803}$ with an N-terminal *GAL4*$_{(768–881)}$ AD-tag in yeast cells, *LEU2*, HA epitope tag	This study
pD153-*kaiC3*-BD	Expression of KaiC3$_{6803}$ with a C-terminal GAL4$_{(1–147)}$ DNA-BD-tag in yeast cells, *TRP1*, c-Myc epitope tag	(4)
pGBK-BD-*kaiC3*	Expression of KaiC3$_{6803}$ with an N-terminal GAL4$_{(1–147)}$ DNA-BD-tag in yeast cells, *TRP1*, c-Myc epitope tag	This study
pCGAD-*kaiA*-AD	Expression of KaiA$_{6803}$ with a C-terminal *GAL4*$_{(768–881)}$ AD-tag in yeast cells, *LEU2*, HA epitope tag	(4)
pGAD-AD-*kaiA*	Expression of KaiA$_{6803}$ with an N-terminal *GAL4*$_{(768–881)}$ AD-tag in yeast cells, *LEU2*, HA epitope tag	This study
pD153-*kaiA*-BD	Expression of KaiA$_{6803}$ with a C-terminal GAL4$_{(1–147)}$ DNA-BD-tag in yeast cells, *TRP1*, c-Myc epitope tag	(4)
pGBK-BD-*kaiA*	Expression of KaiA$_{6803}$ with an N-terminal GAL4$_{(1–147)}$ DNA-BD-tag in yeast cells, *TRP1*, c-Myc epitope tag	This study
pCGAD-*kaiB3*-AD	Expression of KaiB3$_{6803}$ with a C-terminal *GAL4*$_{(768–881)}$ AD-tag in yeast cells, *LEU2*, HA epitope tag	This study
pGAD-AD-*kaiB3*	Expression of KaiB3$_{6803}$ with an N-terminal *GAL4*$_{(768–881)}$ AD-tag in yeast cells, *LEU2*, HA epitope tag	This study
pCGAD-*kaiC1*-AD	Expression of KaiC1$_{6803}$ with a C-terminal *GAL4*$_{(768–881)}$ AD-tag in yeast cells, *LEU2*, HA epitope tag	(4)
pGAD-AD-*kaiC1*	Expression of KaiC1$_{6803}$ with an N-terminal *GAL4*$_{(768–881)}$ AD-tag in yeast cells, *LEU2*, HA epitope tag	(4)
pD153-*kaiC1*-BD	Expression of KaiC1$_{6803}$ with a C-terminal GAL4$_{(1–147)}$ DNA-BD-tag in yeast cells, *TRP1*, c-Myc epitope tag	(4)
pGBK-BD-*kaiC1*	Expression of KaiC1$_{6803}$ with an N-terminal GAL4$_{(1–147)}$ DNA-BD-tag in yeast cells, *TRP1*, c-Myc epitope tag	This study
pCGAD-*kaiB1*-AD	Expression of KaiB1$_{6803}$ with a C-terminal *GAL4*$_{(768–881)}$ AD-tag in yeast cells, *LEU2*, HA epitope tag	This study
pGAD-AD-*kaiB1*	Expression of KaiB1$_{6803}$ with an N-terminal *GAL4*$_{(768–881)}$ AD-tag in yeast cells, *LEU2*, HA epitope tag	This study
pD153-*kaiB1*-BD	Expression of KaiB1$_{6803}$ with a C-terminal GAL4$_{(1–147)}$ DNA-BD-tag in yeast cells, *TRP1*, c-Myc epitope tag	This study

Plasmid Name	Description	Reference
pGBK-BD-*kaiB1*	Expression of KaiB1 with an N-terminal $GAL4_{(1–147)}$ DNA-BD-tag in yeast cells, *TRP1*, c-Myc epitope tag	This study
pGEX-*$kaiA_{6803}$*	Expression of $KaiA_{6803}$ with an N-terminal $GST\text{-}tag_{(1\text{-}231)}$ in *E.coli* cells	(5)
pGEX-*kaiB1*	Expression of KaiB1 with an N-terminal $GST\text{-}tag_{(1\text{-}231)}$ in *E.coli* cells	This study
pGEX-*kaiB3*	Expression of KaiB3 with an N-terminal $GST\text{-}tag_{(1\text{-}231)}$ in *E.coli* cells	This study
pGEX-*kaiC*	Expression of KaiC with an N-terminal $GST\text{-}tag_{(1\text{-}231)}$ in *E.coli* cells	(6)
pGEX-*kaiC3*	Expression of KaiC3 with an N-terminal $GST\text{-}tag_{(1\text{-}231)}$ in *E.coli* cells	(5)
pGEX-*kaiC3-AA*	Can be used for expression of KaiC3-AA with an N-terminal $GST\text{-}tag_{(1\text{-}231)}$ in *E.coli* cells. Was only used for cloning in this study	This study
pGEX-*kaiC3-DE*	Can be used for expression of KaiC3-DE with an N-terminal $GST\text{-}tag_{(1\text{-}231)}$ in *E.coli* cells. Was only used for cloning in this study	This study
pGEX-*kaiC3-catE1⁻catE2⁻*	Can be used for expression of KaiC3- $catE1^{-}catE2^{-}$ with an N-terminal $GST\text{-}tag_{(1\text{-}231)}$ in *E.coli* cells. Was only used for cloning in this study	This study
pASK-kaiC	Expression of KaiC with an N-terminal $Strep\text{-}tag_{(1\text{-}11)}$ in *E.coli* cells	(7)
pASK-kaiC3	Expression of KaiC with an N-terminal $Strep\text{-}tag_{(1\text{-}11)}$ in *E.coli* cells	This study
pASK-kaiC3-*AA*	Expression of KaiC-AA with an N-terminal $Strep\text{-}tag_{(1\text{-}11)}$ in *E.coli* cells	This study
pASK-kaiC3-*DE*	Expression of KaiC-DE with an N-terminal $Strep\text{-}tag_{(1\text{-}11)}$ in *E.coli* cells	This study
pASK-kaiC3-*$catE1^{-}catE2^{-}$*	Expression of KaiC3 -$catE1^{-}catE2^{-}$ with an N-terminal $Strep\text{-}tag_{(1\text{-}11)}$ in *E.coli* cells	This study

Table S2. Plasmids used in this study.

Table S2 references

1. Rausenberger J, Tscheuschler A, Nordmeier W, Wust F, Timmer J, Schafer E, Fleck C, Hiltbrunner A. 2011. Photoconversion and nuclear trafficking cycles determine phytochrome A's response profile to far-red light. Cell 146:813-25.
2. Hiltbrunner A, Viczian A, Bury E, Tscheuschler A, Kircher S, Toth R, Honsberger A, Nagy F, Fankhauser C, Schafer E. 2005. Nuclear accumulation of the phytochrome A photoreceptor requires FHY1. Curr Biol 15:2125-30.
3. Shimizu-Sato S, Huq E, Tepperman JM, Quail PH. 2002. A light-switchable gene promoter system. Nat Biotechnol 20:1041-4.

4. Axmann IM, Dühring U, Seeliger L, Arnold A, Vanselow JT, Kramer A, Wilde A. 2009. Biochemical evidence for a timing mechanism in *Prochlorococcus*. J Bacteriol 191:5342-7.

5. Wiegard A, Dörrich AK, Deinzer HT, Beck C, Wilde A, Holtzendorff J, Axmann IM. 2013. Biochemical analysis of three putative KaiC clock proteins from *Synechocystis* sp. PCC 6803 suggests their functional divergence. Microbiology 159:948-958.

6. Nishiwaki T, Satomi Y, Nakajima M, Lee C, Kiyohara R, Kageyama H, Kitayama Y, Temamoto M, Yamaguchi A, Hijikata A, Go M, Iwasaki H, Takao T, Kondo T. 2004. Role of KaiC phosphorylation in the circadian clock system of *Synechococcus elongatus* PCC 7942. Proc Natl Acad Sci U S A 101:13927-32.

7. Oyama K, Azai C, Nakamura K, Tanaka S, Terauchi K. 2016. Conversion between two conformational states of KaiC is induced by ATP hydrolysis as a trigger for cyanobacterial circadian oscillation. Sci Rep 6:32443.

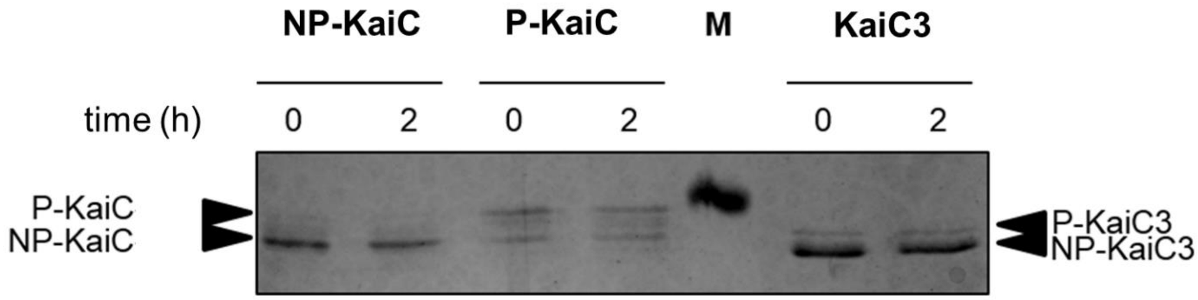

Figure S1: Phosphorylation level of KaiC proteins used for ATP synthase activity assay shown in Fig. 1. Proteins were separated via SDS-PAGE using a polyacrylamide gel with 11 %T, 0.67 %C (see dx.doi.org/10.17504/protocols.io.gysbxwe for method description). Fully phosphorylated (P-KaiC) and dephosphorylated (NP-KaiC) *S. elongatus* KaiC proteins were generated by incubating the protein for 2 weeks at 4 °C or overnight at 30 °C, respectively.

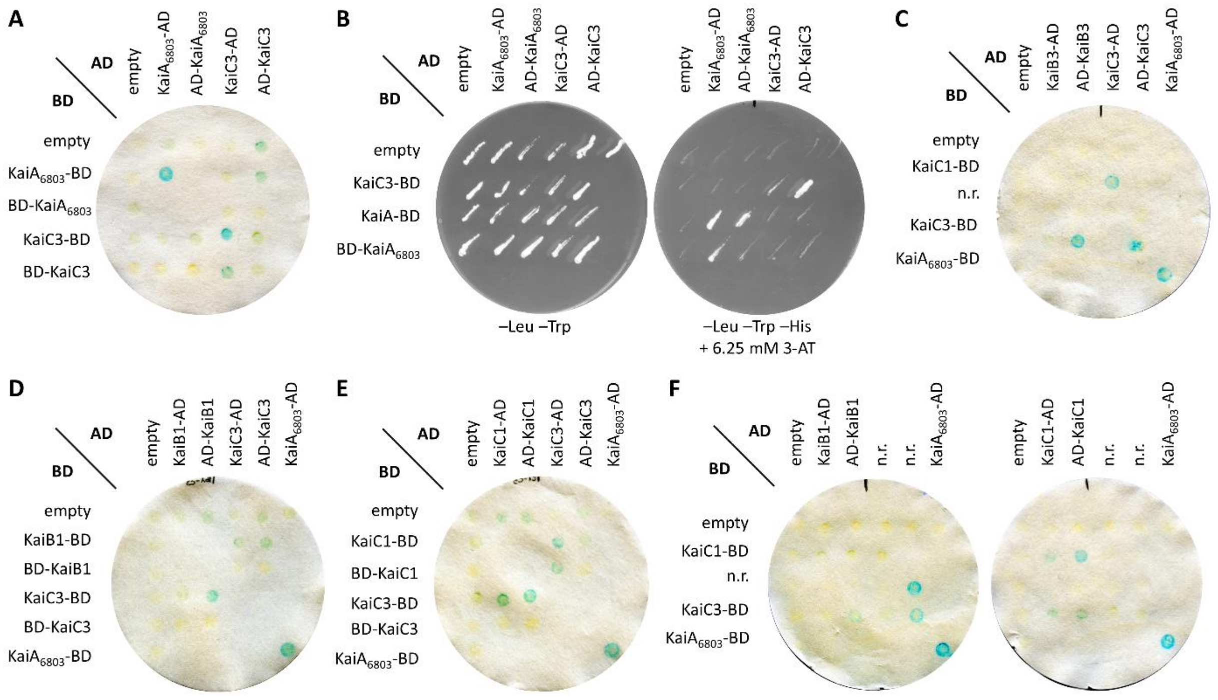

Figure S2. Original scans of the KaiC3 interaction with KaiB3 and the proteins of the main oscillator KaiC1, KaiB1. Yeast two-hybrid reporter strains carrying the respective bait and prey plasmids, were selected by plating on complete supplement medium (CSM) lacking leucine and tryptophan (-Leu -Trp). As a positive control, $KaiA_{6803}$ dimer interaction was used. AD, GAL4 activation domain; BD, GAL4 DNA-binding domain; n.r., interactions not relevant for this study. **A, C-F:** Physical interaction between bait and prey fusion proteins is indicated by a color change in the assays using 5-brom-4-chlor-3-indoxyl-β-D-galactopyranoside. **B:** Physical interaction between bait and prey fusion proteins is determined by growth on complete medium lacking leucine, tryptophan and histidine (-Leu -Trp -His) and addition of 6.25 mM 3-amino-1,2,4-triazole (3-AT). A detailed protocol can be found on protocols.io (https://dx.doi.org/10.17504/protocols.io.wcnfave).

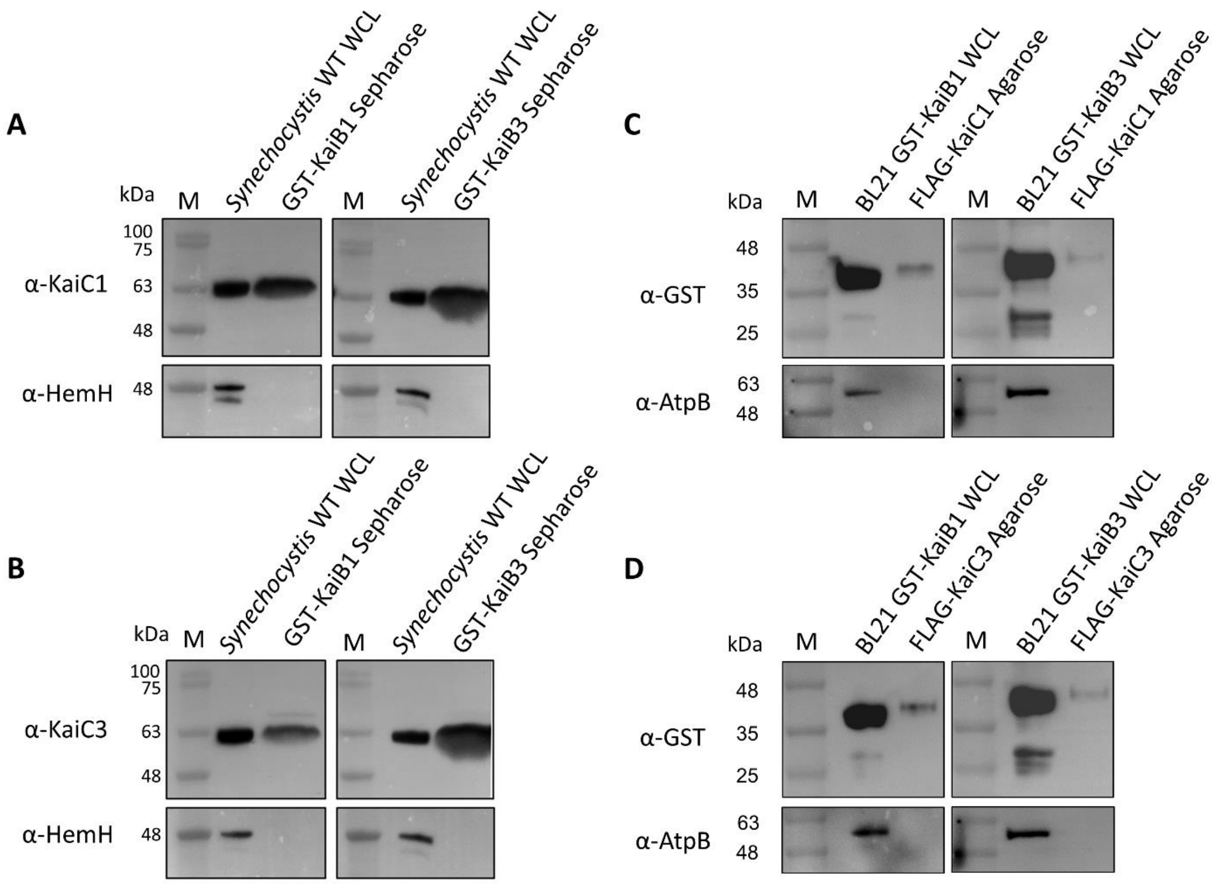

Figure S3. Interaction of *Synechocystis* KaiB and KaiC proteins in pull down analysis. A,B GST-tagged KaiB1 and KaiB3 proteins were expressed in *E. coli* BL21 cells, bound to glutathione sepharose and incubated with *Synechocystis* WT whole cell lysate (WCL). In the eluate, KaiC1 (A) and KaiC3 (B) were detected by Western Blot analysis using specific antibodies. As negative control, blots were incubated with an antiserum against the ferrochelatase HemH, because we did not expect an interaction between the KaiB proteins and HemH. **C,D** FLAG- KaiC1 (C) and FLAG-KaiC3 (D) were expressed in *Synechocystis*, bound to Anti-FLAG-agarose and incubated with whole cell lysate (WCL) from *E. coli* BL21 cells expressing GST-KaiB1 and GST-KaiB3 proteins, respectively. To detect whether GST-KaiB proteins were co-eluted we used an antibody raised against GST. Incubation with an AtpB antibody served as negative control. Both experiments were performed once.

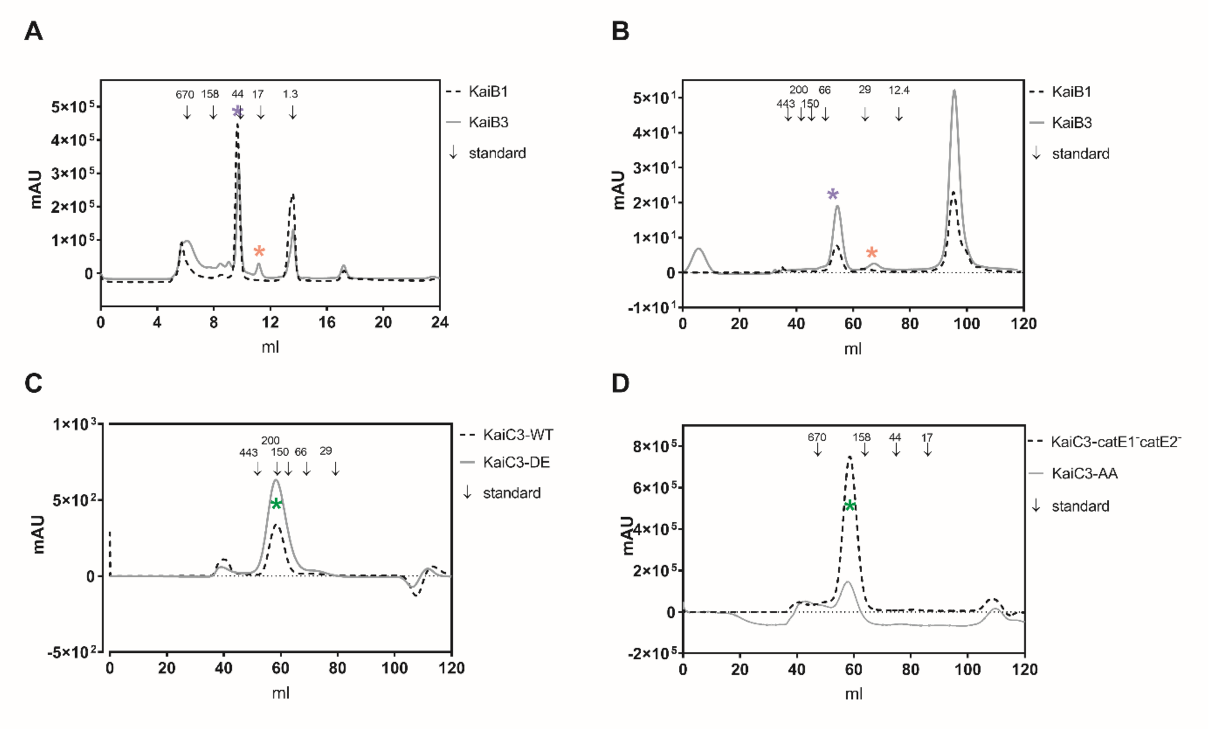

Figure S4: Purification of KaiB and KaiC3 proteins used in this study. AB: KaiB proteins were subsequently purified via affinity chromatography, anion exchange chromatography and size exclusion chromatography. Shown are the chromatograms after size exclusion chromatography using a superdex 200 Increase 10/30 GL column (A) or Sephacryl S200 HR HiPrep 16/60 column (B). On both columns KaiB3 was separated into a monomer (red asterisk) and tetramer (blue asterisk), whereas KaiB1 was mainly eluted as tetramer (blue asterisk). **CD**: KaiC3 proteins were purified via affinity chromatography followed by size exclusion chromatography on a Sephacryl S300 HR HiPrep 16/60 Sephacryl column. All KaiC3 proteins eluted as oligomer (green asterisk). Arrows indicate the size of standard proteins in kDa.

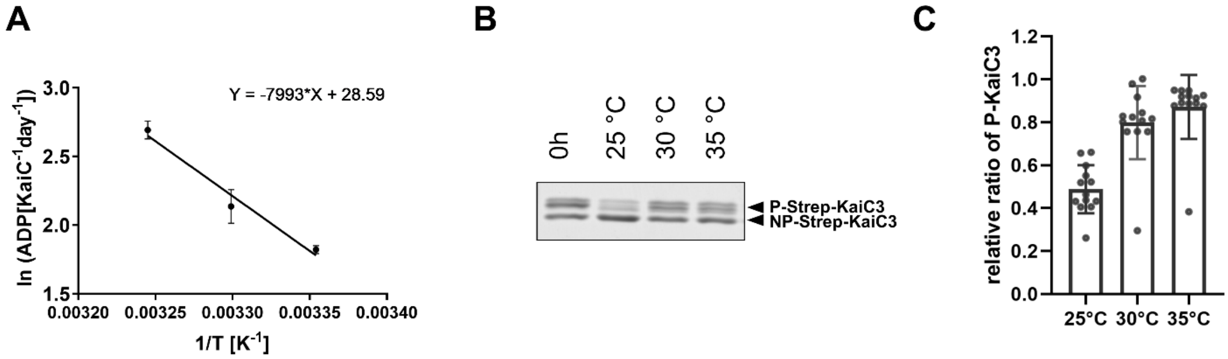

Figure S5. ATPase activity and dephosphorylation of KaiC3 are temperature dependent. A. Temperature-dependent ATPase activity of Strep-KaiC3 (also displayed in Fig. 4B) shown in an Arrhenius plot. Strep-KaiC3 was incubated for 24 hours at 25°C, 30°C and 35°C and ADP production per day and monomer Strep-KaiC3 was calculated. To determine the activation energy, the natural logarithm of the mean ADP production rate was plotted against the reciprocal temperature. The activation energy of KaiC3 ATPase was calculated from the slope of a linear regression equation as E_a=66.5 x kJ mol^{-1}. **B,C.** Relative dephosphorylation of Strep-KaiC3. Strep-KaiC3 was incubated for 24 hours in 20 mM Tris-HCl/pH8, 150 mM NaCl, 5 mM $MgCl_2$, 1 mM ATP at the indicated temperatures. Proteins were separated via SDS-PAGE using a polyacrylamide gel with 11 %T, 0.67 %C. The phosphorylation level at each temperature was determined as the ratio of P-Strep-KaiC3 to total Strep-KaiC3 (P-Strep-KaiC3 + NP-Strep-KaiC3) using ImageJ. A representative gel image is shown in **B**. In **C**, the relative phosphorylation levels compared to the stock protein (0h) of 5 experiments are plotted as mean values with standard deviation. Incubation at 25°C resulted in lower phosphorylation levels, demonstrating higher net-dephosphorylation.
